# Ultrahigh pressure phase transition in ice

**DOI:** 10.1038/s41563-026-02732-1

**Published:** 2026-09-07

**Authors:** Israel Osmond, Mikhail A. Kuzovnikov, David A. Lewis, Hannah A. Shuttleworth, Tomás Marqueño, Milo J. Dixon, Cerian E. A. Robertson, Vitan Cherepnalkoski, Bihan Wang, Andreas Hermann, Miriam Peña-Alvarez, Ross T. Howie

**Affiliations:** 1 Centre for Science at Extreme Conditions, The University of Edinburgh, Edinburgh, UK; 2 School of GeoSciences, The University of Edinburgh, Edinburgh, UK; 3 Deutsches Elektronen-Synchrotron DESY, Hamburg, Germany; 4 Center for High Pressure Science and Technology Advanced Research, Shanghai, China

**Keywords:** Structure of solids and liquids, Phase transitions and critical phenomena, Phase transitions and critical phenomena

## Abstract

One of the most enduring problems in dense H_2_O ice has been the experimental realization of the pressure-induced symmetry breaking of cubic ice X to an orthorhombic crystal structure. Here, through diamond anvil cell experiments combined with synchrotron X-ray diffraction and Raman spectroscopy techniques, we demonstrate that post-symmetrization, the crystal structure of ice X continuously distorts with pressure. Subjecting ice to pressures in excess of 308(5) GPa, we observe a first-order phase transition to ice XXII, consistent with the long-predicted phase possessing an orthorhombic crystal structure with *Pbcm* symmetry. We find that ice XXII is stable to pressures of at least 340(5) GPa.

## Abstract (web summary)

The crystal structure of ice X is observed to distort under extreme pressure at room temperature before transforming to ice XXII, the most dense H_2_O polymorph experimentally observed.

## Main

Ice exists in the most extreme environments of the universe, from comets travelling through the cold vacuum of space, to the hot, dense interiors of giant planets. Terrestrial experiments continue to discover new forms of ice at high pressure–temperature conditions^1–9^, such as superionic states, which bridge the stability fields of solid and fluid water^5,10^. Concurrently, state-of-the-art theoretical works predict a rich landscape of unexplored structural phases of ice, which seemingly are beyond the current technical limitations of high-pressure experimentation^11–17^.

Despite exhibiting a complex pressure–temperature phase diagram, upon room-temperature compression, the phase sequence of ice is relatively simple, with only three known solid phases (VI, VII, X), all of which have been extensively studied^18–21^. Ices VII and X share the same body-centred cubic crystal lattice of oxygen atoms and only differ in the arrangement of their hydrogen atoms^22^. Upon compression, the isolated H_2_O molecules of phase VII become indistinguishable, transforming to hydrogen-ordered phase X, within which hydrogen atoms are midway between nearest-neighbour oxygen atoms^23–28^.

For almost 30 years, density functional theory (DFT) calculations have predicted that cubic ice X should transform to an orthorhombic crystal structure (with *Pbcm* symmetry) at higher pressures^12,17,28–30^. This orthorhombic modification of crystalline ice is predicted to be stable over a vast pressure regime, exceeding that of any other experimentally observed phase^8,12,17^. The transition to *Pbcm* ice has never been experimentally realized, and, up until now, remained one of the most important unresolved problems in the most abundant molecule on Earth.

Despite the importance for planetary science, the studies of H_2_O at such ultrahigh pressures are limited owing to the experimental challenge. This is due to a combination of constrained small sample volumes, weak Raman scattering cross section and ice inherently being a poor X-ray scatterer. Nevertheless, vast improvements have been made in diagnostic techniques, namely the brilliance of synchrotron sources, combined with the focusing capabilities at X-ray diffraction beamlines^31,32^.

To investigate the properties of ice at ultrahigh pressure conditions, we have performed a series of static compression experiments, reaching pressures in excess of 330 GPa. Through synchrotron X-ray diffraction and Raman spectroscopy measurements, we demonstrate that post-symmetrization of hydrogen bonds, the crystal lattice of ice X continuously distorts upon compression. Above 308(5) GPa, we observe a first-order phase transition from distorted ice X to ice XXII, which is consistent with the previously predicted *Pbcm* phase.

### Compression of ice X

**Fig. 1:**
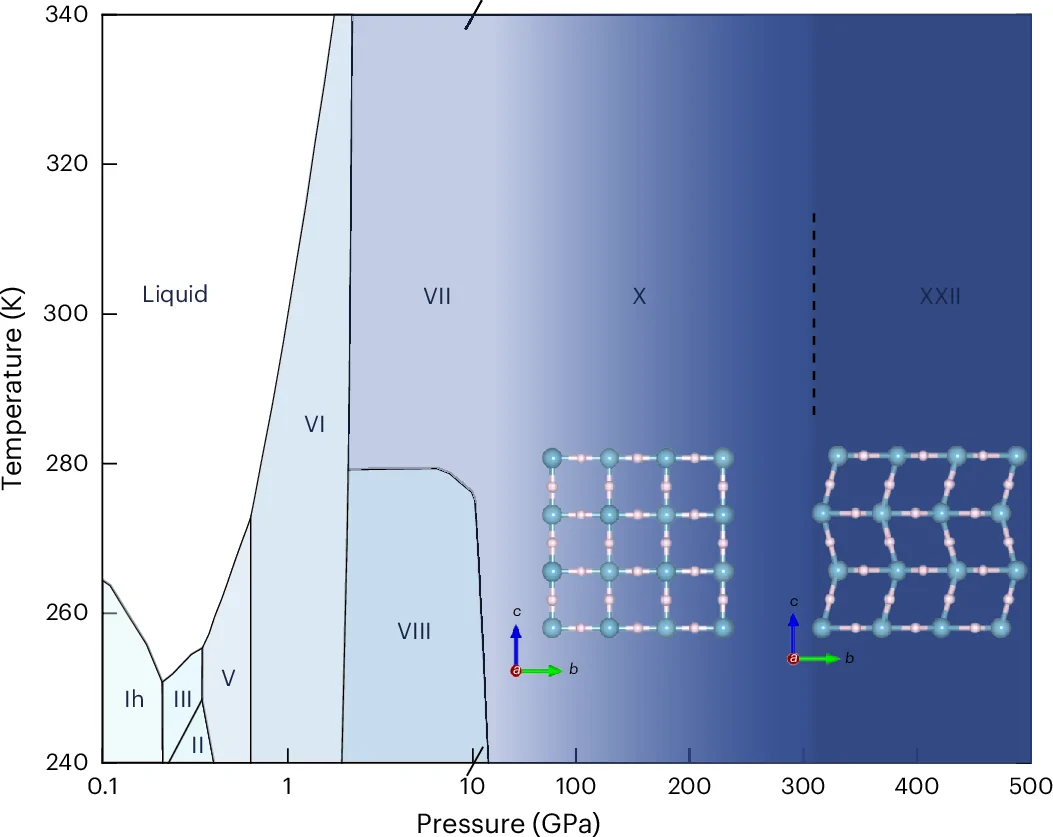
Proposed phase diagram of ice up to 500 GPa. Phase lines shown in black are taken from ref. ^42^ and references therein. The dashed line represents the transition pressure to phase XXII. Inset: structure of cubic ice X (space group $Pn\overline{3}m$) and ice XXII (space group *Pbcm*).

The molecular structures of all currently known ices obey the so-called H_2_O ice rules—each oxygen atom is bound to four neighbouring oxygen atoms by hydrogen bonds, and each bond is realized by one hydrogen atom^2,5,33–36^. At room temperature, fluid H_2_O crystallizes into ice VI with *P*4_2_/*nmc* space group at 1.0 GPa, before transforming to ice VII with $Pn\bar{3}m$ symmetry at 2.2 GPa (refs. ^37,38^) (see phase diagram in Fig. 1). At pressures above 70 GPa, ice VII continuously transforms to ice X and it is indistinguishable from a supercritical crossover^25,26,39^. Ices VII and X share the same body-centred cubic crystal lattice of oxygen atoms and only differ in the arrangement of their hydrogen atoms^22,40^.

While the transition from ice VII to ice X is unidentifiable in X-ray diffraction experiments, the Raman spectra provide clear signatures of the changing crystal symmetry, arising from shifts in hydrogen atomic positions. These changes are not necessarily centred on the phase boundary or Widom line but are nonetheless characteristic of the phase change^39^. Upon compression, it has been previously shown that the symmetric stretching mode of H_2_O ice VII is not observed above 60 GPa, while above 80 GPa the translational modes are superseded by a single band in phase X^26^. According to group theory analysis, there is only one allowed Raman active phonon (T_2g_) in $Pn\bar{3}m$ H_2_O ice X, corresponding to the vibrations of oxygen atoms surrounded by stationary hydrogen atoms^26,28^.

**Fig. 2:**
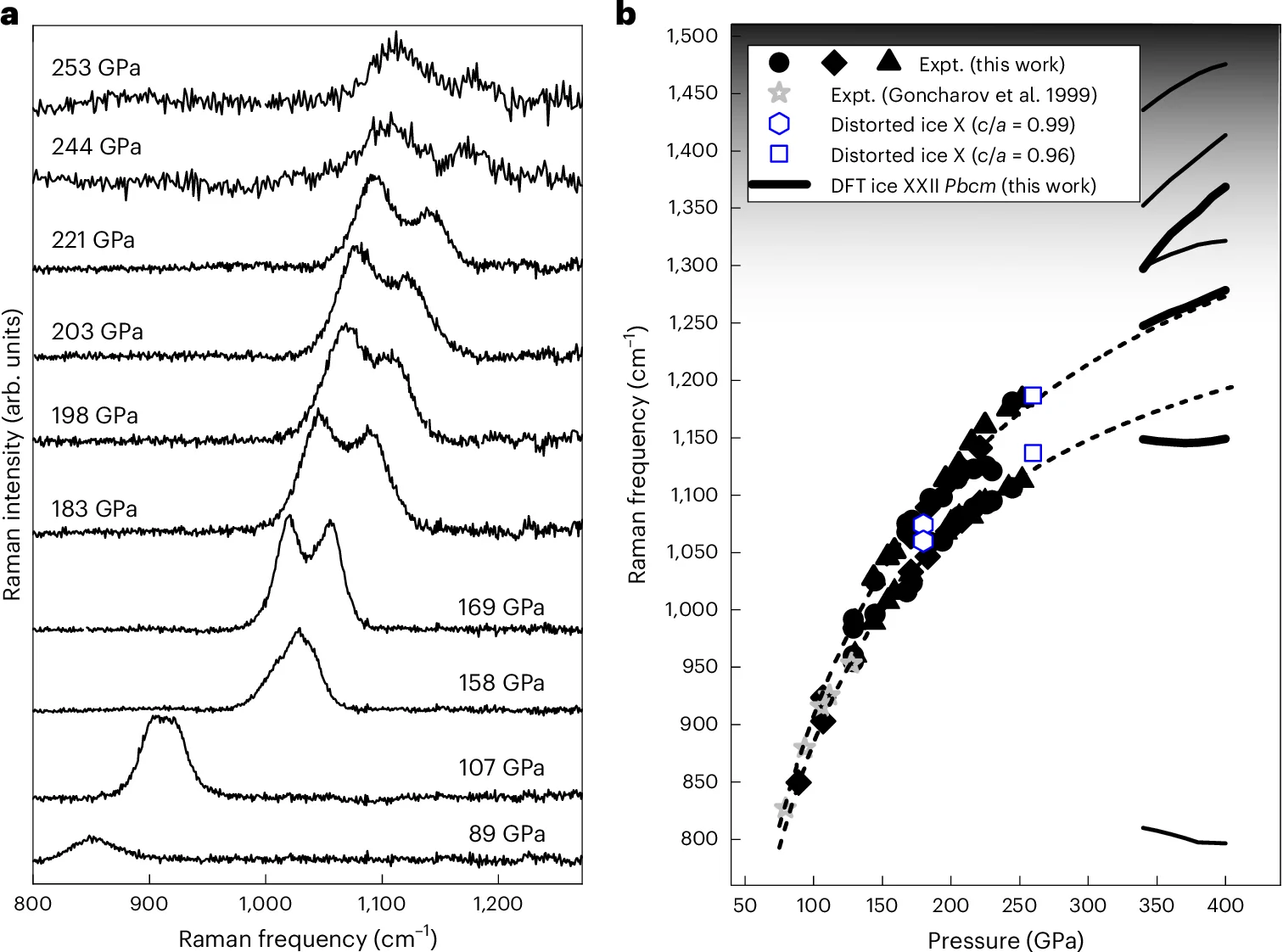
Representative Raman spectra and frequency shift upon compression. **a**, Representative Raman spectra upon compression to 253 GPa. **b**, Raman frequency as a function of pressure of the modes observed in ice X. Filled symbols represent the frequencies taken from fitting two modes to the experimental data. Different symbols represent different experimental runs. Black dashed lines represent extrapolations of the experimental data as a guide to the eye. Grey open stars represent the previously reported experimental frequencies of ice X^26^. The open blue symbols represent the calculated shifts for tetragonally distorted $Pn\bar{3}m$ ice X (Methods), and black solid lines represent the DFT calculated modes of *Pbcm* ice XXII with thicker lines used for the most intense modes. The grey shaded area represents the frequency regime where the first-order Raman band from the diamond anvil would obscure the sample bands. The gradient represents the tail of the diamond band.

**Fig. 3:**
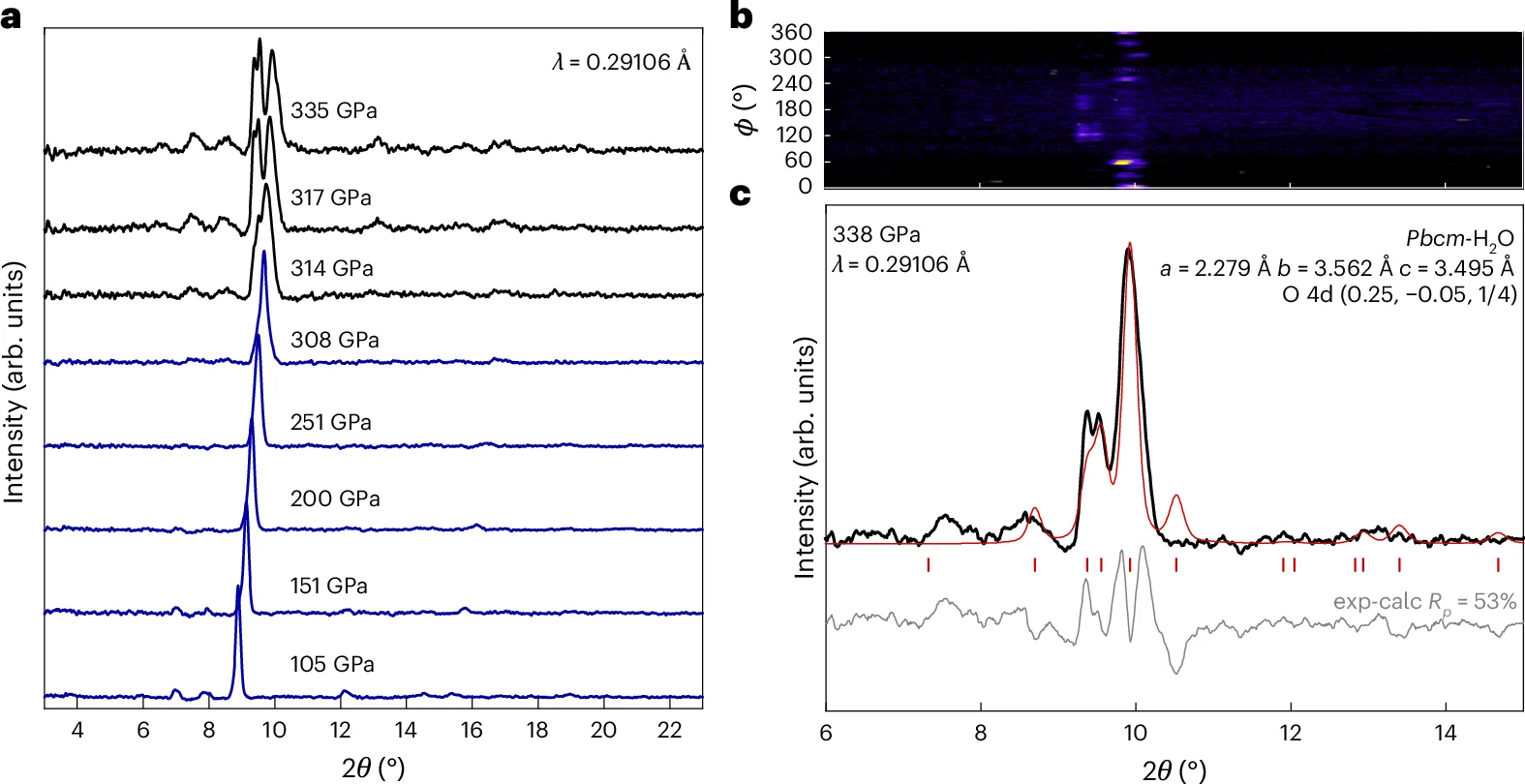
Representative X-ray diffraction patterns upon compression and Rietveld refinement of ice XXII at 338 GPa. **a**, Representative X-ray diffraction patterns upon compression from 105 GPa to 335 GPa. Ices X and XXII are represented by dark blue and black, respectively. Further diffraction patterns can be found in the Supplementary Information. **b**, 2D diffraction image of phase XXII at 338 GPa. **c**, Rietveld refinement of ice XXII (*Pbcm*) at 338 GPa. The black, red and grey curves show the experimental structure factor, the Rietveld fit and the fit residual, respectively. The position of the oxygen atom was taken from DFT calculations at 400 GPa (Supplementary Table 1) and fixed.

We have conducted high-resolution Raman spectroscopy measurements of ice X up to pressures of 253 GPa (Fig. 2). We observe the appearance of the T_2g_ mode above 80 GPa, which is in agreement with a previous study that tracked the frequency dependency up to a maximum pressure of 128 GPa (ref. ^26^). However, we find that the T_2g_ mode continues to broaden until it splits into two distinct and resolvable modes above 160 GPa. While both modes harden with pressure, the splitting increases (for example, Δ*ν* ≃ 52 cm^−1^ and 69 cm^−1^ at 167 GPa and 253 GPa, respectively). This was observed in all experimental runs and the frequency of each mode at a given pressure was consistent between experiments. The splitting of the T_2g_ mode is indicative of a change in the symmetry of the oxygen lattice. Concomitantly, the X-ray diffraction measurements in all experimental runs demonstrate a marked asymmetry in the (110) reflection of ice X at pressures above ~100 GPa (Fig. 3a and Supplementary Figs. 1, 5, 10 and 11), coinciding with the splitting of the T_2g_ Raman mode. We attribute this to a weak tetragonal distortion of the crystal lattice. Unfortunately, within this pressure regime, the (200) and (211) reflections are too weak to discern any asymmetry.

Within calculations, any distorted structures of ice X are mechanically unstable below 300 GPa and relax back to cubic ($Pn\bar{3}m$) symmetry. In fact, a mechanical instability (*c*_12_ > *c*_11_) of the $Pn\bar{3}m$ model for ice X only develops around 500 GPa, well within the energetic stability region of the predicted *Pbcm* phase (see Supplementary Fig. 2 for the calculated elastic stiffness coefficients of ice X). This instability leads to a tetragonal variant of ice X (space group *P*4_2_/*nnm*) that has been predicted recently as a metastable phase^17^. As the only available candidate structure, we have used the *P*4_2_/*nnm* structural model in the Rietveld refinements above 100 GPa to account for a possible distortion. While the predicted phase is elongated along *c* at 500 GPa (*c*/*a* ~1.20), our measurements find a small compression of *c*, which increases with pressure (for example, *c*/*a* ~0.99 at 140 GPa to *c*/*a* ~0.97 at 300 GPa). We have simulated Raman spectra of different tetragonally distorted variants of ice X at a series of densities. We find good agreement between the experimental and calculated Raman frequencies at the experimentally determined distortions (Fig. 2 and Supplementary Fig. 3). Very similar behaviour is seen in NH_4_F, an ionic ice analogue, where NH_4_F-III (a structural analogue of ice X) develops a tetragonal distortion across a wide pressure range, before transforming to a new high-pressure phase^41^. Similarly, it would seem that this tetragonal distortion is intrinsic to ice X and not due to strain effects within the sample chamber. An assessment of whether these observations could be attributed to deviatoric stress conditions of the sample is included in the Supplementary Information.

### Transition to ice XXII

Upon further compression, we observe distorted ice X up to 308 GPa, above which there are distinct changes in the X-ray diffraction pattern (Fig. 3a). Over a pressure regime of ~10 GPa, we observe further splitting of the first ice X reflections into 3 distinct components, indicative of a transformation to another polymorph, henceforth called ice XXII.

The presence of three distinct components in the main group of peaks suggests that the symmetry of ice XXII is no higher than orthorhombic. The higher-angle peak at 10° is more intense than the lower-angle peaks. This indicates that the orthorhombic distortion is applied to the $1\times \sqrt{2}\times \sqrt{2}$ supercell of the cubic structure rather than to the original cubic unit cell, which would otherwise result in a splitting into three components with equal intensities. Such a distortion corresponds to an *Abmm* space group. In some of the ice XXII patterns (such as that shown in Supplementary Fig. 5), we observe a weak feature at 10.5°, which could be indexed as (021). Among the maximal subgroups of *Abmm*, only *Pbcm* and *Pbmb* allow the presence of the (021) reflection. *Pbmb* can be rejected on the grounds that it should also have an appreciable intensity of the (001) peak at 4.7°, which is not observed experimentally. Thus, the *Pbcm* space group provides the highest symmetry compatible with the experimentally observed X-ray diffraction patterns of ice XXII.

DFT-based computational quantum chemistry has previously predicted that H_2_O should transform into an orthorhombic structure with *Pbcm* space group upon compression above 250–350 GPa (refs. ^12,17,28–30^). Figure 3c shows a comparison of the crystal structure factor of ice XXII at 338 GPa with the simulated structure factor of the predicted *Pbcm* model, relaxed with DFT at 400 GPa, with the refined lattice parameters *a* = 2.279 Å, *b* = 3.562 Å and *c* = 3.495 Å. Despite the weak sample signal and strong grain effect, we observe a good agreement regarding the intensity distribution in the main peak group. The intensities of the (100), (110) and (021) reflections of the *Pbcm* model, expected at 7.3°, 8.7° and 10.5°, respectively, depend on the atomic coordinates of the oxygen atom, O (0.251, −0.054, 1/4), which were taken from the DFT calculation at 400 GPa. These coordinates were fixed in the Rietveld refinement to directly compare the structure factor of the predicted *Pbcm* model with the experiment. Refining these coordinates does not significantly improve the residual *R*-factors, which are mostly limited by the grain effect.

**Fig. 4:**
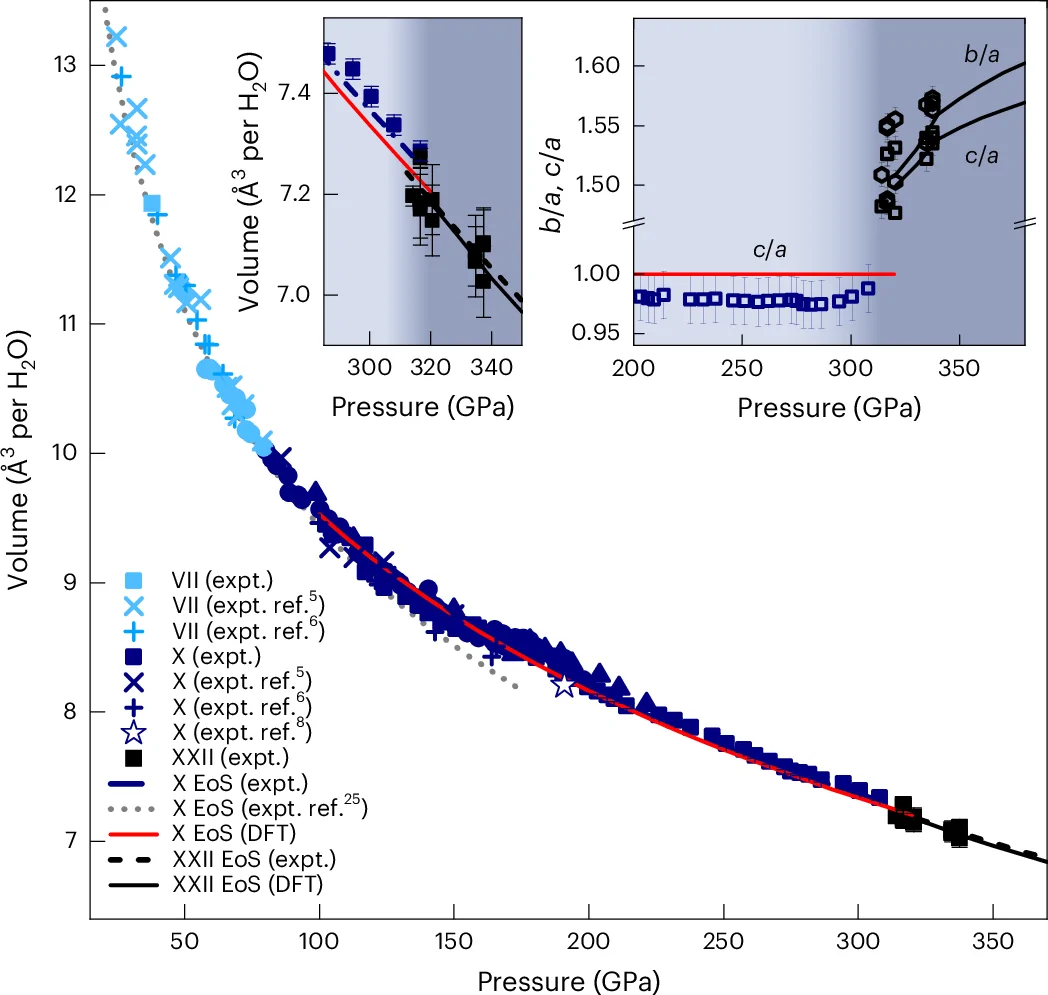
Volume as a function of pressure for the observed ice phases. Light blue symbols, dark blue symbols and black symbols represent phases VII, X and XXII, respectively. The dashed dark blue line corresponds to the Birch–Murnaghan fits of phases X (between 108 GPa and 308 GPa) and the dashed black line is a Birch–Murnaghan fit to phase XXII. The results of the DFT *P*–*V* equations of state (EoS) of $Pn\bar{3}m$ phase X and *Pbcm* phase XXII are shown as red and black solid lines, respectively. The grey dotted line shows the previously reported EoS of ice to 170 GPa (ref. ^25^). The cross, plus and open star symbols represent experimental data from refs. ^5,6,8^, respectively. Left inset: zoomed view of the volume as a function of pressure at the ice X to XXII transition region. Right inset: the *b*/*a* (hexagon symbols) and *c*/*a* ratios (square symbols) for phase X (dark blue) and phase XXII (black). The solid red and black lines represent the *b*/*a* and *c*/*a* ratios for the DFT phases X and XXII, respectively. The error bars represent the model uncertainty in the Rietveld analysis of X-ray diffraction data, which mostly originates from the refinement of the peak full-width at half-maximum and preferred orientation.

There is also good agreement regarding the magnitude of the orthorhombic splitting (right inset in Fig. 3), and the pressure at which the peak splits, which allows us to assign a *Pbcm* crystal structure model to ice XXII. Structurally, the O–H–O bonds remain the same in *Pbcm* compared with ice X, but adjacent layers in the *ab* plane are sheared with respect to each other (inset in Fig. 1). Both the structure and transition pressure are in good agreement with previous predictions^12,17,28–30^. Upon the transition to ice XXII, we observe a discontinuity in volume of −0.04 Å^3^ per H_2_O, which is consistent with our DFT calculated value of −0.02 Å^3^ per H_2_O and indicative of a first-order phase transition (Fig. 4). In one experimental run, pressure dropped from 338 GPa to ~39 GPa owing to the failure of one of the diamond anvils, which resulted in a transformation back into ice VII (Supplementary Fig. 7).

To assess possible non-hydrostatic effects, we have compared the X-ray diffraction patterns collected at each pressure from the sample and the gold pressure marker (Supplementary Figs. 4–6 and 8). As gold is surrounded by the sample, the possible non-hydrostatic effects should affect both patterns. Particularly, if the observed peak splitting in the X-ray diffraction patterns of the sample at around 308 GPa is to be associated with non-hydrostaticity, the comparable changes should be additionally observed in the gold pattern. However, no pressure-induced changes in the profile of the gold reflections are observed, which rules out such an interpretation.

In a subsequent experimental run, we were able to reproduce the transition to ice XXII. However, the data quality was much poorer, giving more data scatter in the extracted volumes, with the pressure overestimated owing to the gold calibrant being compressed against the gasket. This data can be found in Supplementary Figs. 9, 10 and 12. Taking this overestimation into account, this experimental run demonstrated ice XXII to be stable to at least 355(10) GPa.

We were unable to measure the Raman spectra of ice XXII owing to the weak Raman scattering of H_2_O, combined with increased pressure-induced fluorescence engulfing the sample signal in the low frequency regime at pressures above 250 GPa. However, extrapolating the Raman frequencies of the modes observed in distorted ice X, the values are consistent with two of the most intense modes calculated through DFT of *Pbcm* ice (Fig. 2 and Supplementary Figs. 3 and 14). This further suggests that the distorted ice X is an intermediary state leading to phase XXII. While complementary Raman and IR spectroscopy studies exploring the ice X to XXII transition could provide invaluable information, inherent limitations of current diamond anvil cell experiments would hinder such studies. In the case of Raman studies, all but one of the most intense Raman modes for the *Pbcm* structure are in a frequency regime that would be obscured by the stressed diamond anvil (grey shaded area in Fig. 2b). Although the IR stretching band of ice X is predicted to split upon the transition to ice XXII, the most intense modes lie at extrapolated frequencies of ice X and would most probably be indiscernible (Supplementary Figs. 3, 15 and 16).

Ice XXII is predicted to be stable over a vast range of pressure–temperature space, with another orthorhombic modification, with *Pbca* symmetry, expected to emerge at 760 GPa (ref. ^11^). However, this is currently beyond the experimental limitations of conventional static high-pressure experiments and poses the next generational challenge. Shock studies have shown that at temperatures exceeding 2,000 K and in the pressure regime where we observe phase XXII, ice adopts an fcc structure, ice XVIII^3^. Bridging the gap between static and dynamic compression experiments to understand the XXII–XVIII transition, as well as how these relate to the superionic states, will be the subject of great future interest.

In summary, we observe a distortion of ice X above pressures of 160 GPa, leading to the formation of ice XXII, at 308(5) GPa; to our knowledge, this is the most dense H_2_O polymorph ever observed (Fig. 1). Ice XXII possesses an orthorhombic crystal structure, which is in agreement with quantum chemistry calculations predicting a *Pbcm* phase^29^. Our study is an advancement towards understanding the internal structure of giant planets, and provides evidence that the state-of-the-art quantum chemistry calculations based on DFT accurately reproduce the behaviour of H_2_O under such extreme environments.

## Methods

### Sample preparation and pressure calibration

We conducted a total of 10 independent experimental runs up to a maximal pressure of 355 ± 5 GPa. Two of these experimental runs exceeded pressures required for the transition to phase XXII. Pressure was generated using a custom-designed (piston–cylinder type SH) symmetric diamond anvil cell. These cells were equipped with Boehler-Almax cut diamonds from Almax-easyLab (type IIas for X-ray diffraction, type IIam for Raman spectroscopy), with culet diameters ranging from 15 μm to 50 μm bevelled to a diameter of 200 μm. We used either Al_2_O_3_ composite gaskets or rhenium gaskets. The Al_2_O_3_ composite gaskets were created by initially indenting 200 μm rhenium foil to a thickness of ~6 μm. The foil was then drilled using a custom-built UV laser micromachining system with a hole diameter slightly larger than the culet diameter. An Al_2_O_3_-epoxy powder was then placed in the indent and compressed until a thickness of ~6 μm was achieved. The sample chamber was then drilled using the UV laser micromachining system to a diameter approximately 50% culet size. In other experiments, rhenium foils were indented to a thickness ranging between 5 μm and 10 μm, and sample chambers drilled to a diameter approximately 50% culet size. In all experiments, high-purity Milli-Q H_2_O was loaded. No reaction was observed between the rhenium or the Al_2_O_3_ composite gasket and H_2_O over the pressure regime studied.

In X-ray diffraction studies, a small amount of gold powder was placed inside the sample chamber, and pressure was determined using the previously established volume (pressure) equation of state of gold^43^. In Raman spectroscopy measurements, the pressure was determined using the frequency of the stressed edge of the diamond anvil. The first-order diamond Raman band becomes elongated in frequency space with pressure. The frequency of the stressed culet was determined by the frequency (*ν*) at which *dI*/*dν* was minimized (where *I* is the intensity of the mode). Pressure was calculated using the calibration given in ref. ^44^.

### X-ray diffraction measurements

Angular-dispersive powder X-ray diffraction experiments above pressures of 250 GPa were performed at the Extreme Conditions Beamline (P02.2) at PETRA III, Hamburg, Germany^32^. Additional data below 250 GPa were collected at the ID15B beamline at the ESRF, Grenoble, France^31^. Typically, the diamond anvil cell was oscillated around the *ω* axis by either ±5° or ±10°, and the 2D diffraction image was collected with acquisition times ranging between 60 s and 240 s.

At P02.2, we used an incident X-ray beam with *E* ≈ 42.4 keV (*λ* ≈ 0.292 Å), focused to a 2 μm × 2 μm spot using Kirkpatrick–Baez mirrors. Diffraction images were recorded using a PerkinElmer XRD1621 detector with a sample-to-detector distance (SDD) of ~400 mm, as calibrated with a CeO_2_ standard. At ID15B, an incident beam with *E* ≈ 30.2 keV (*λ* ≈ 0.410 Å) was focused to an approximately 1 μm × 1 μm spot. The diffraction patterns were collected with an EIGER2 X 9M detector with SDD ≈ 158 mm. The SDD was calibrated using a Si powder standard.

The SDD, detector orientation and wavelength calibration, primary processing, azimuthal integration and first background subtraction were performed with the DIOPTAS v0.6.1 software^45^. All detector images collected upon compression from each individual cell were processed with the same mask to enable common background subtraction at a later stage. At pressures of about 350 GPa, the ratio of the maximum diffraction signal to the background owing to Compton scattering from diamonds was between 1/50 and 1/300 depending on the experimental run. Spatial variations of the detector counting efficiency produced additional intensity variations of about 1/200. To correct for these, we manually subtracted a common background from each pattern after proper exposure normalization (Supplementary Fig. 17). Rietveld refinements were done with the POWDERCELL 2.4 programme^46^ and FULLPROF 7.00. Only the first peak group in the patterns, resulting from the splitting of the (110) reflection from ice X, was clearly visible up to the highest pressure, and for this reason, only the peaks from this group were used for the refinements. For the *Pbcm* model, only the lattice parameters and, in some cases, the March parameter for the March–Dollase (100) preferred orientation model were refined, whereas the atomic parameters were taken from DFT and fixed to directly compare the structure factor of the predicted *Pbcm* model with the experiment. Atomic displacement parameters were not refined owing to the narrow useful range of momentum transfers.

### Raman spectroscopy measurements

A custom-built Raman spectroscopy setup was used to collect Raman spectra in a confocal backscattering geometry. We used a 514.53 nm Ar-laser (Innova 70C, Coherent), with a typical output power of 20 mW, as the excitation source. An IsoPlane 320 spectrometer (Princeton Instruments) equipped with 1,200 mm^−1^ and 1,800 mm^−1^ gratings and a liquid nitrogen cooled PyLoN CCD (Princeton Instruments) was used. Before collecting each spectrum, the spectrometer was calibrated using an Ar–Ne lamp. Typical spectra were collected with acquisition times of 60 s and 10 accumulations.

### DFT calculations

We performed DFT calculations with the CASTEP code^47^ using norm-conserving pseudopotentials. A plane wave cut-off of 1,200 eV and a Monkhorst–Pack^48^ *k*-point grid with spacing 0.04 Å^−1^ was sufficient to give enthalpy differences between ice X and *Pbcm,* which were converged to better than 1 meV per formula unit. Of the different exchange-correlation functionals tested (BLYP^49^, PBE^50^, PBEsol^51^, revPBE^52^ and revPBE+D4 (refs. ^52–55^)), we found that the BLYP functional *P*(*V*) equation of state aligns best with the experimental data for ices X and XXII, and was used for all phonon, IR and Raman calculations. For a given pressure, BLYP has the largest cell volume, and revPBE+D4 the smallest. The predicted transition pressure from ice X to *Pbcm* varies by almost 50 GPa across all functionals considered. The calculated X → XXII transition pressures are approximately 260 GPa, 300 GPa, 320 GPa, 330 GPa and 340 GPa for PBEsol, PBE, revPBE, revPBE+D4 and BLYP, respectively. We further find a ground state transition from the proton-ordered ice VIII to ice X of ~130 GPa with BLYP. Standard DFT calculations are known to overestimate the pressure onset of stability for ice X owing to additional quantum effects that are not accounted for here^24,39^. Phonon frequencies and IR/Raman intensities were obtained with CASTEP within the density functional perturbation theory framework^56^. Phonon density of states calculations used a *q*-point Monkhorst–Pack grid spacing of 0.08 Å^−1^ and a fine interpolation grid with 0.02 Å^−1^ spacing. Elastic stiffness coefficients were obtained from linear stress–strain relationships derived from appropriate lattice strains of magnitudes ±0.5% and ±1%, and subsequent relaxation of atomic positions (for example, see refs. ^57–59^). Raman spectra for tetragonally distorted ice X were obtained for several pressure points, along a series of volume-conserving tetragonal distortions of the ice X unit cells that covered the range *c*/*a* ≈ 0.85–1.15.

## Online content

Any methods, additional references, Nature Portfolio reporting summaries, source data, extended data, supplementary information, acknowledgements, peer review information; details of author contributions and competing interests; and statements of data and code availability are available at https://doi.org/10.1038/s41563-026-02732-1.

## Supplementary information

Supplementary Information An analysis of the stress condition of the sample environment, together with Supplementary Tables 1 and 2 and Figs. 1–18.

## Data Availability

The X-ray diffraction data is available in the University of Edinburgh DataShare repository (ref. ^60^), and Raman spectroscopy data will be made available upon request.
