# Supplementary material for: Ultrahigh pressure phase transition in ice
Source: Nat Mater. 2026 Sep 7;25(10):1850–4. doi: 10.1038/s41563-026-02732-1 (PMC13619449; doi:10.1038/s41563-026-02732-1)
Supplement: Supplementary file 1 [file 41563_2026_2732_MOESM1_ESM.pdf]

nature materials

Supplementary information

https://doi.org/10.1038/s41563-026-02732-1

Ultrahigh pressure phase transition in ice

In the format provided by the authors and unedited

Contents

1 Analysis of the stress conditions of the sample environment 2

2 Supplementary Tables 3

3 Supplementary Figures 6

1 Analysis of the stress conditions of the sample environment

If x-ray diffraction data is collected over a broad angular 2θ range then the deviatoric stress can be estimated through two approaches – one based on the systematic diffraction peak shift [1, 2]; and another one based on the peak broadening [3]. In the case of our H_2O samples, both of these methods are inapplicable because in the pressure range of interest, the useful diffraction signal from our samples is only observed in a narrow angular range 8-11° (compared with 11-38° in Ref. [2]).

However, we can apply these methods to the x-ray diffraction measurements of the gold pressure marker, which is embedded within the sample chamber, under the assumption that both the sample and pressure marker are under the same deviatoric stress conditions.

Using the approach of Ref. [3] based on the angular dependence of FWHM, we have individually fitted each diffraction peak of the strained gold with a pseudo-Voigt function (except (200), which overlaps with ice), and the resulting FWHM were plot in appropriate coordinates in Fig. S8b. At each individual pressure the observed FWHM dependencies were fitted with the linear function, and the resulting slope gave us the strain 4ε, which was then plotted as a function of pressure in Fig. S8c. The resulting dependence is rather weak and do not show any noticeable change upon the transition to ice XXII at 308 GPa.

Additionally, we tried to adopt the peak shift approach of Refs. [1, 2] for the x-ray diffraction pattern of gold at 200 GPa. The Gamma plot for this pattern is shown below in Fig. S8d along with a linear fit; as one can see, this linear fit has a negligible slope comparing to the data scatter, which indicates that the peak shifts in our case are not due to the strain, and have different origins such as stacking faults.

2 Supplementary Tables

Phase	Pressure	Lattice	Atomic positions
X	260 GPa	$a = 2.480$ Å	O $2a(1/4, 1/4, 1/4)$
($Pn\bar{3}m$)			H $4c(1/2, 1/2, 1/2)$
XXII	400 GPa	$a = 2.237$ Å	O $4d(0.2519, -0.0549, 1/4)$
($Pbcm$)		$b = 3.544$ Å	H1 $4d(-0.0003, 0.6969, 1/4)$
		$c = 3.482$ Å	H2 $4b(1/2, 0, 0)$

Supplementary Table 1. Crystallographic information for $Pn\bar{3}m$ ice X and $Pbcm$ ice XXII, from DFT-BLYP calculations at representative pressures as listed.

Substance	Pressure range (GPa)	EoS type	V_0 (Å^3/H_2O)	B_0 (GPa)	B'_0	Source
ice VII/X	4–128	Birch–Murnaghan	20.4(5)	23.7(9)	4.15(7)	[4]
ice X	63–126	Vinet	13.37(22)	145(12)	4 (fixed)	[5]
ice VII/X	2.5–170	Vinet	24.11	4.26	7.75	[6]
ice X	157–308	Birch–Murnaghan	12.9	174	4 (fixed)	present
ice XXII	314–338	Birch–Murnaghan	16.6	70	4 (fixed)	present

Supplementary Table 2. Equation of state parameters for high-pressure ice phases

3 Supplementary Figures

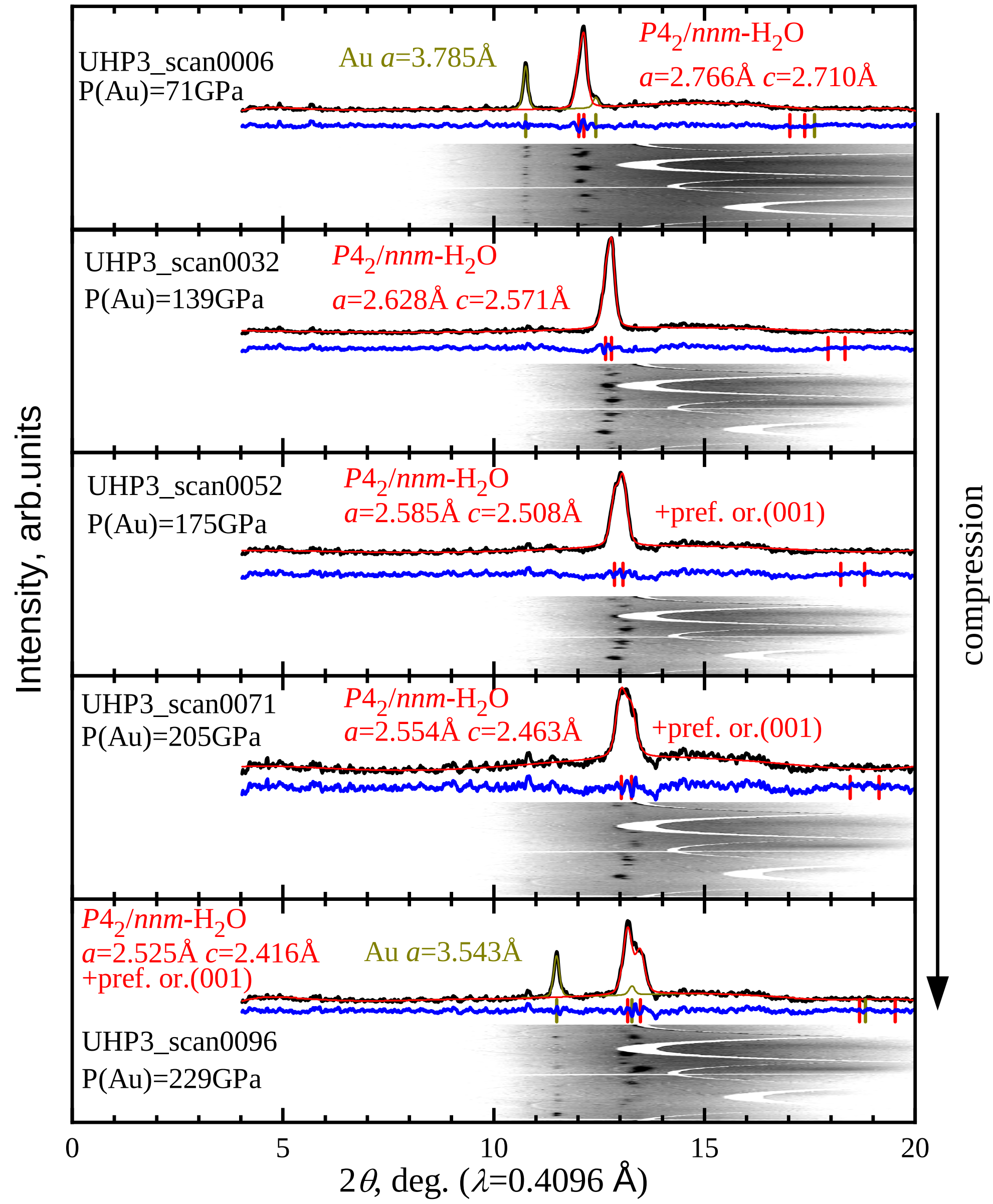

Supplementary Figure 1. Representative x-ray powder diffraction patterns of H_2O (black curves) at 71, 139, 175, 205 and 229 GPa, together with the results of the Rietveld refinements for the structural model for $P4_2/nnm$ (red). Experimental data are from the experimental run UHP3 . The refinement residuals are shown in blue. The Au contributions shown as dark yellow curves in the refinement. Inserts below the residuals are the corresponding 2D diffraction images.

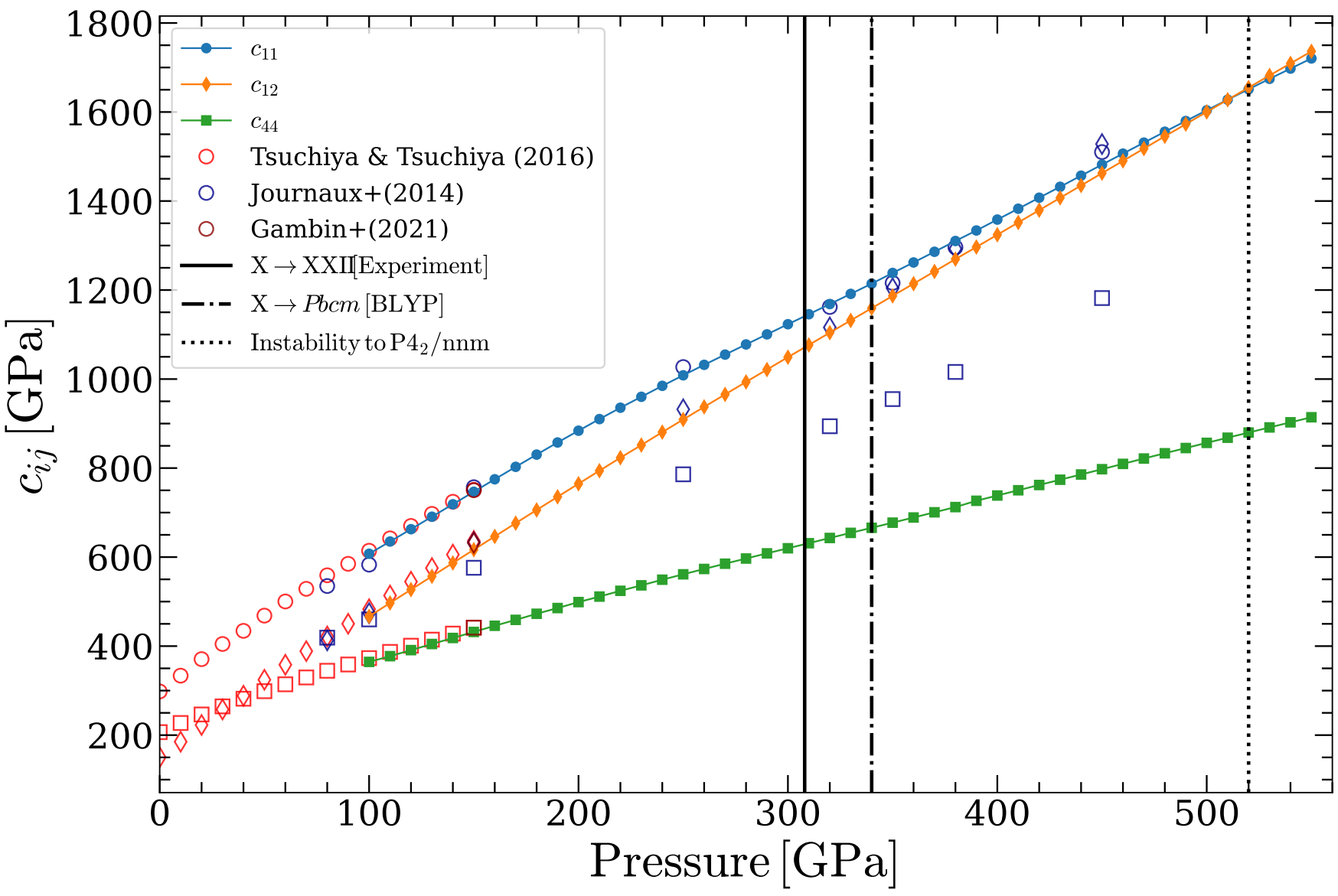

Supplementary Figure 2. Calculated elastic stiffness coefficients c_{11} (circle), c_{12} (diamond), and c_{44} (square) for ice X between 100 and 550 GPa. Solid symbols are calculated in this work (see Methods), open symbols are from previous computational studies [7–9]. The solid black line corresponds to the experimentally observed transition pressure for ice X to ice XXII and the dashed-dot line corresponds to pressure onset of stability of the *Pbcm* phase over $Pn\bar{3}m$ ice X in DFT (see Supplementary Figure 13). The mechanical instability ($c_{12} > c_{11}$) in $Pn\bar{3}m$ ice X occurs at 520 GPa and is shown with the black dotted line.

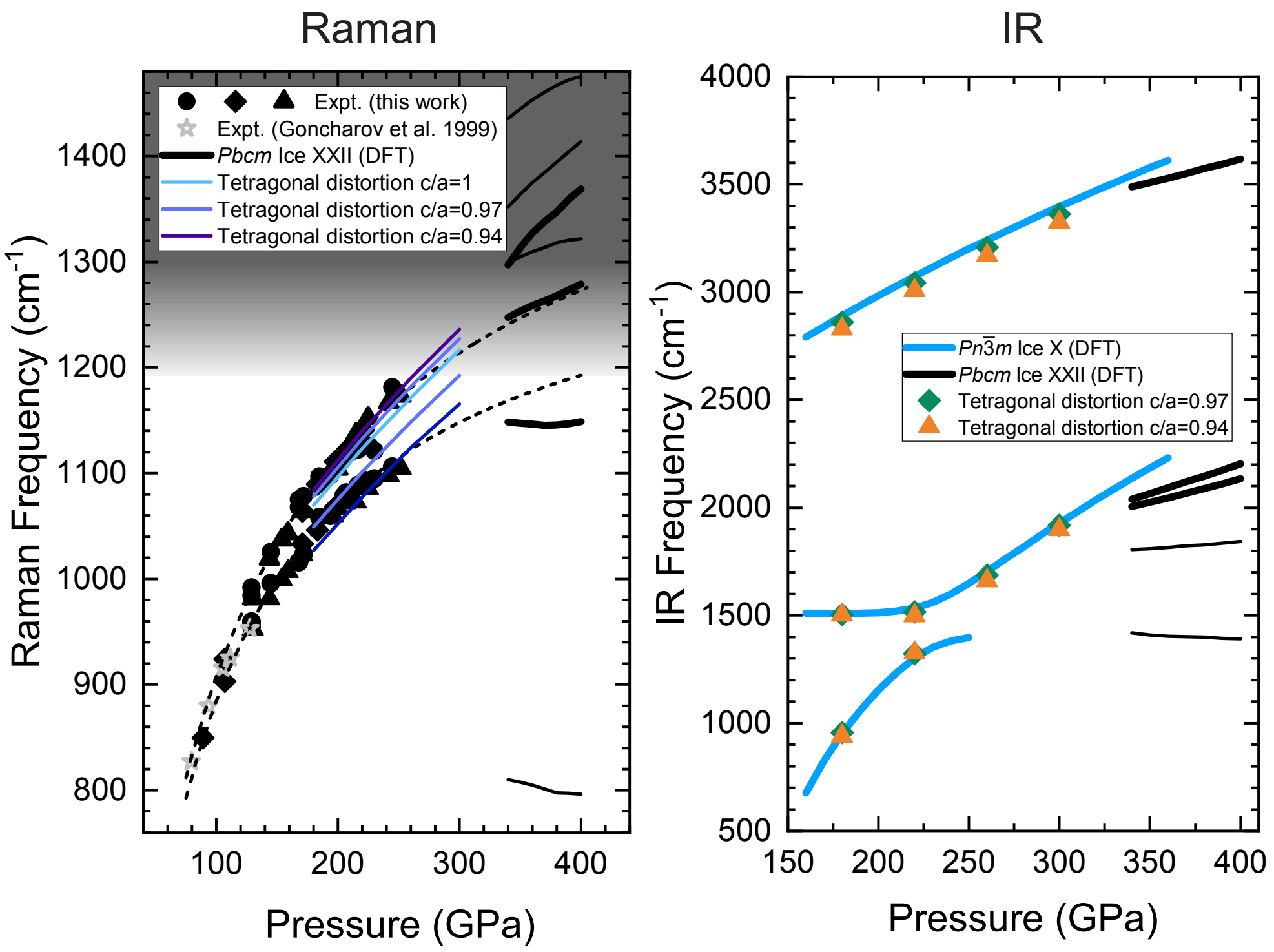

Supplementary Figure 3. Left: Raman frequency as a function of pressure of the modes observed in Ice X. Solid symbols represent the frequencies taken from fitting two modes to the experimental data. Different symbols represent different experimental runs. Black dashed lines represent extrapolations of the experimental data as a guide to the eye. Grey open stars represent the previously reported experimental frequencies of ice X [10]. The light blue line is undistorted $Pn\bar{3}m$, dark blue lines and purple lines are for distorted $Pn\bar{3}m$ with c/a ratios of 0.97 and 0.94 respectively, and black lines represents the DFT calculated modes of *Pbcm* ice XXII, with the thickest lines correspond to the most intense bands, see Figure S8 for full Raman spectra. The grey shaded area represents the frequency regime where the first order Raman band from the diamond anvil would obscure the sample bands. The gradient represents the tail of the diamond band. Right: DFT calculated infrared frequencies for: undistorted $Pn\bar{3}m$ ice X as light blue lines and tetragonal distorted $Pn\bar{3}m$ with c/a of 0.97 and 0.94 are shown as green diamonds and orange triangles. IR DFT calculated frequencies of *Pbcm* ice XXII are shown as black lines. Full IR spectra are presented at Figures S9 and S10.

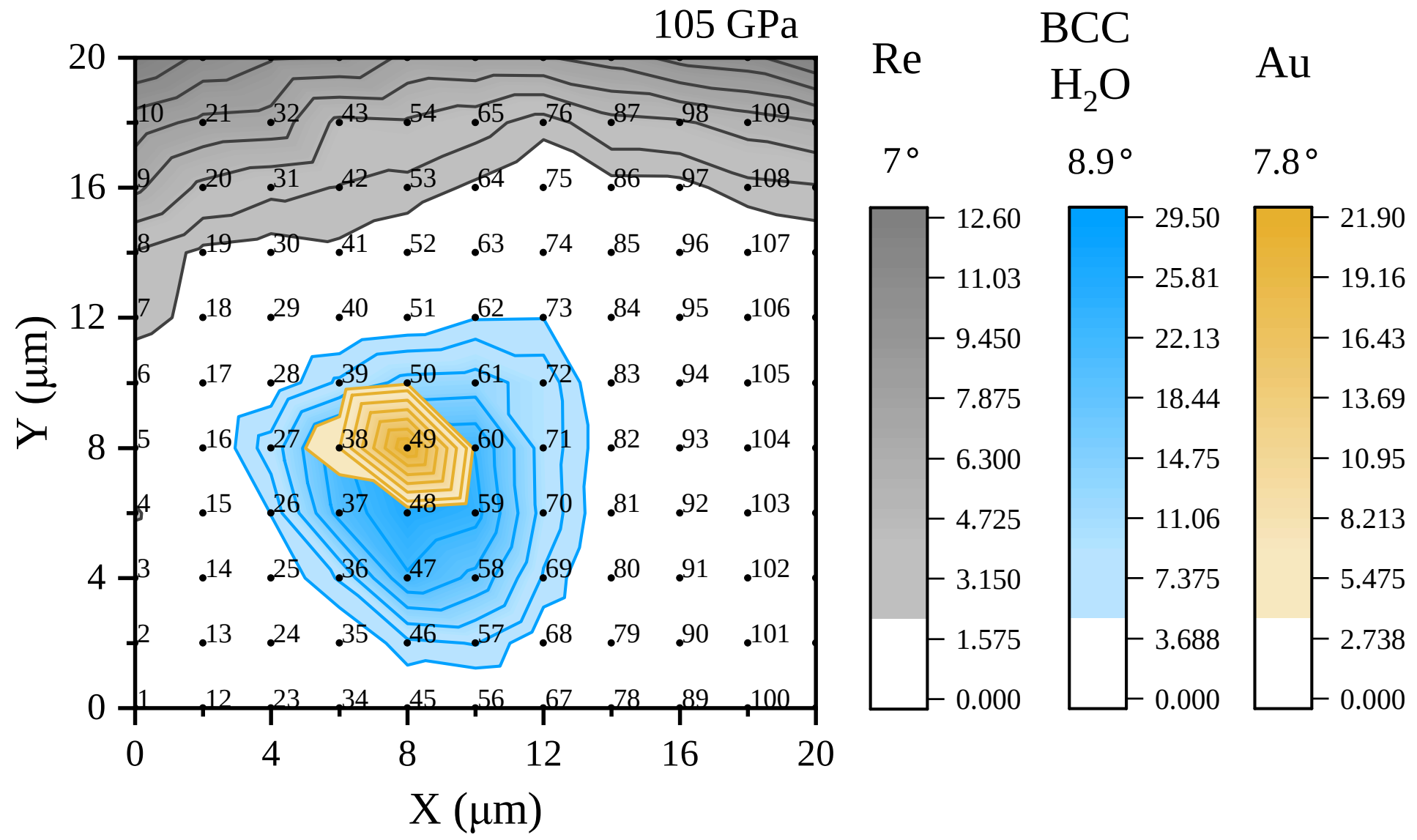

Supplementary Figure 4. Contour plot of the XRD mapping of the sample chamber, 1 μm step size, from the experimental run UHP8, corresponding to the data in Supplementary Figure 5. Blue regions indicate diffraction from ice where the (110) reflections of ice X are identified. Yellow regions correspond to diffraction peaks from Au, where the (111) reflections. Data were collected using a monochromatic X-ray beam with wavelength $\lambda = 0.29163$ Å.

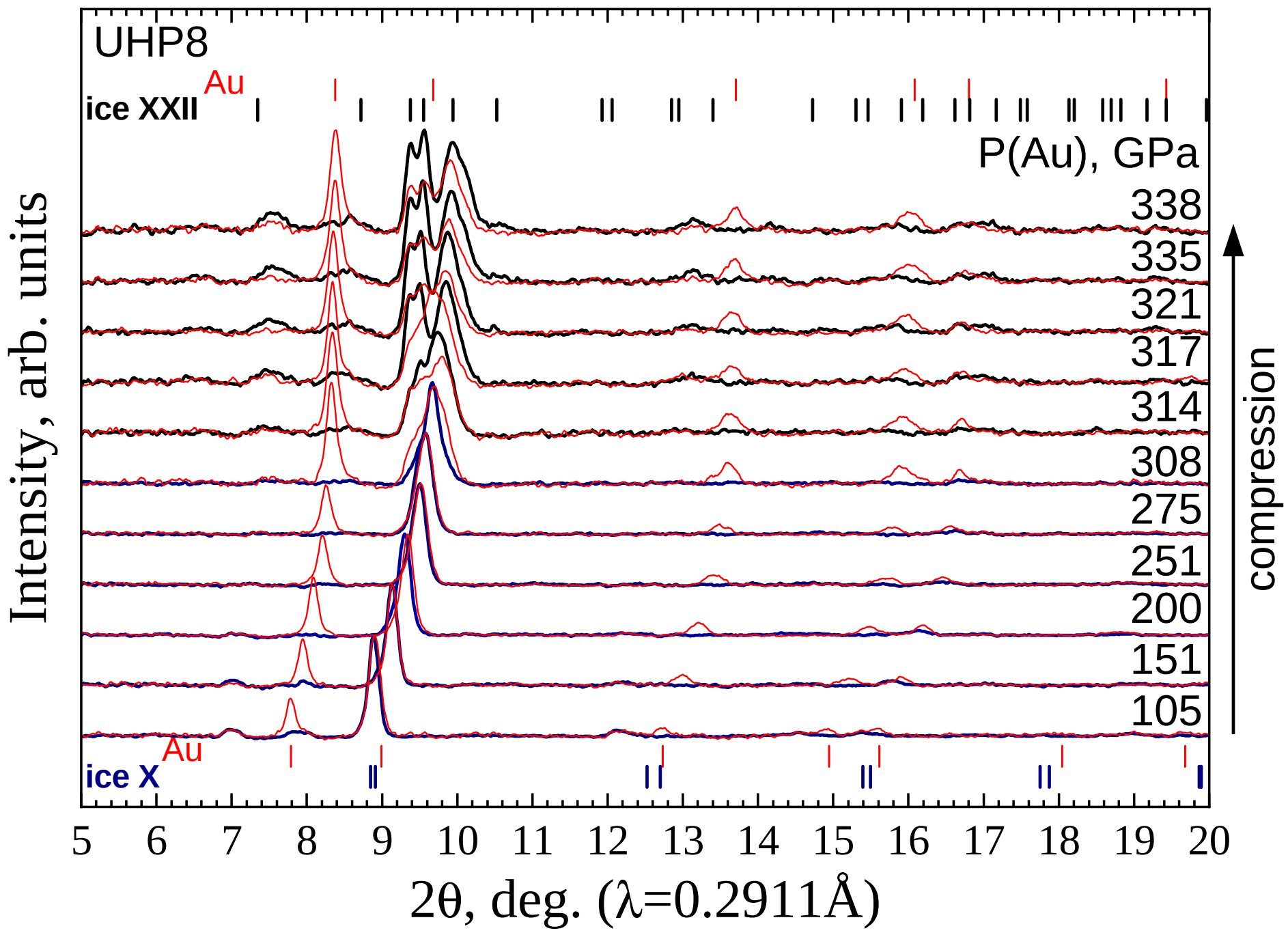

Supplementary Figure 5. Representative x-ray diffraction patterns upon compression from 105 GPa to 338 GPa, from experimental run UHP8. Dark blue patterns correspond to ice X, whilst black patterns correspond to phase XXII. The diffraction patterns from the gold pressure standard are overlayed in red. Pressures are calculated from the gold equation of state [11].

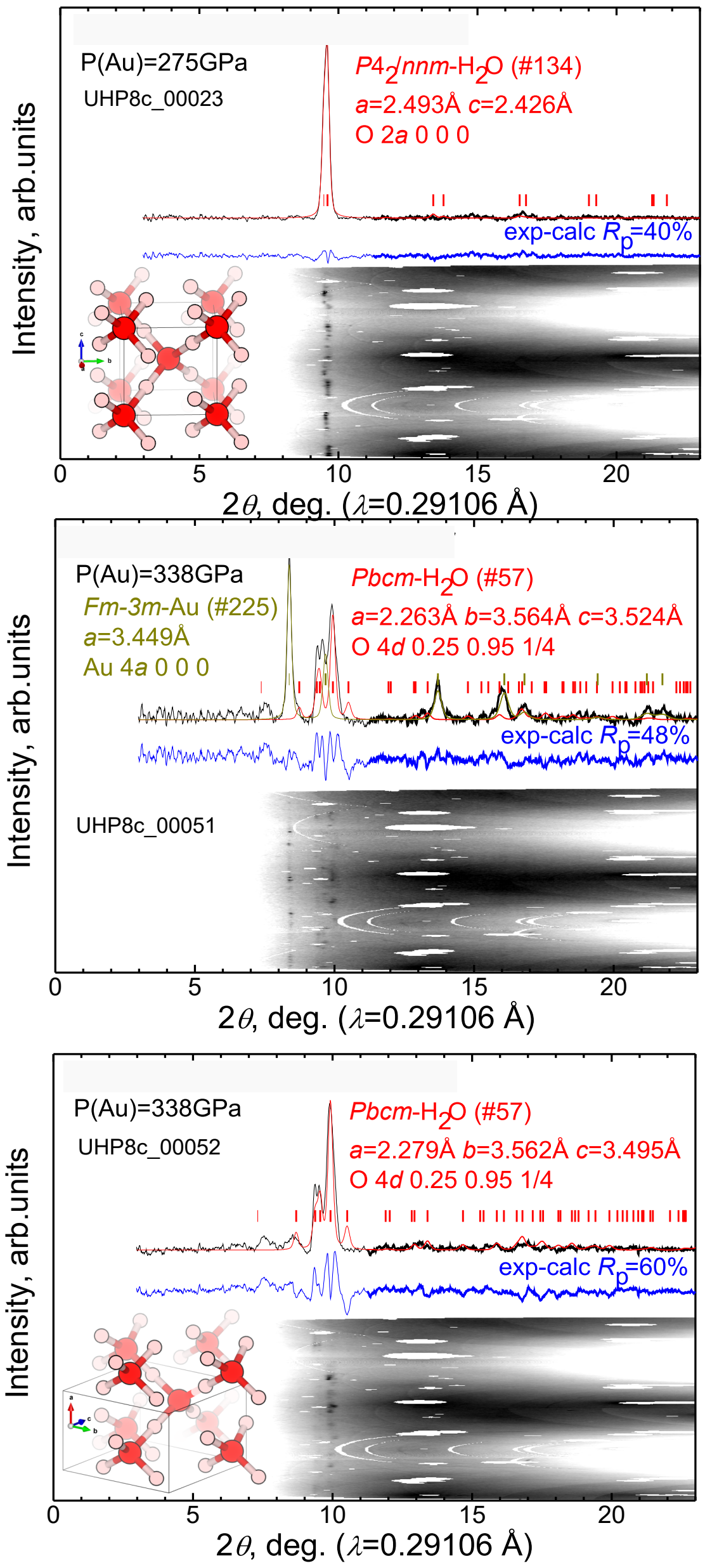

Supplementary Figure 6. Representative x-ray powder diffraction patterns of H_2O (black curves) at 275 and 338 GPa, together with the results of the Rietveld refinements (red) of the structural model for $P4_2/nnm$, at 275 GPa, and $Pbcm$ at 338 GPa. Experimental data are from the experimental run UHP8 and are shown in black. At 338 GPa we show two patterns collected at two different sample positions, with the gold pressure marker (middle) and without (bottom). The refinement residuals are shown in blue. Inserts below the residuals are the corresponding 2D diffraction cake images.

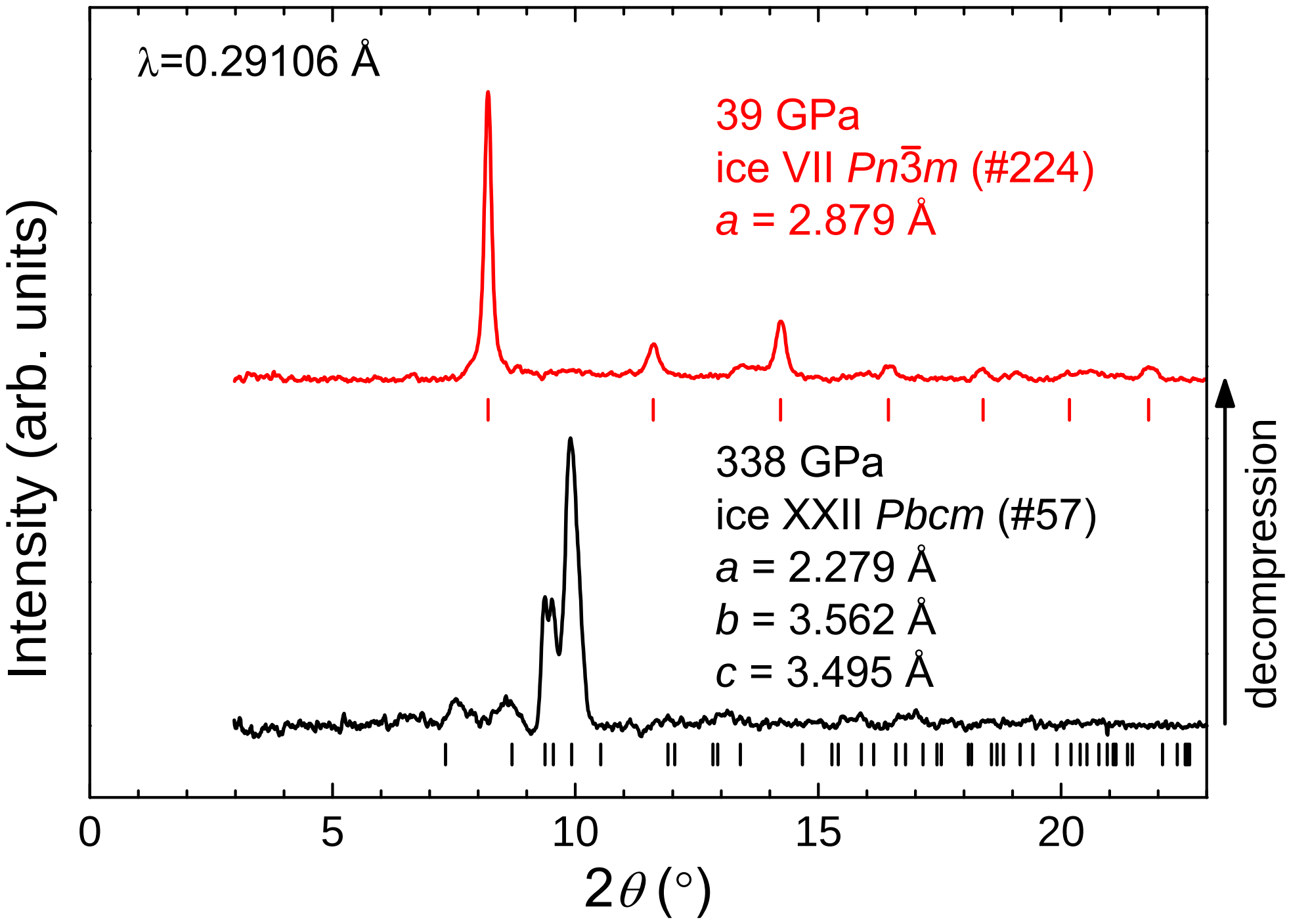

Supplementary Figure 7. Representative x-ray diffraction patterns at 335 GPa (ice XXII) and upon decompression to 38 GPa (ice VII) directly after anvil failure from experimental run UHP8. This is demonstrative the transition is reversible and the observed changes upon compression are due to phase transitions in ice and not due to chemical reactions.

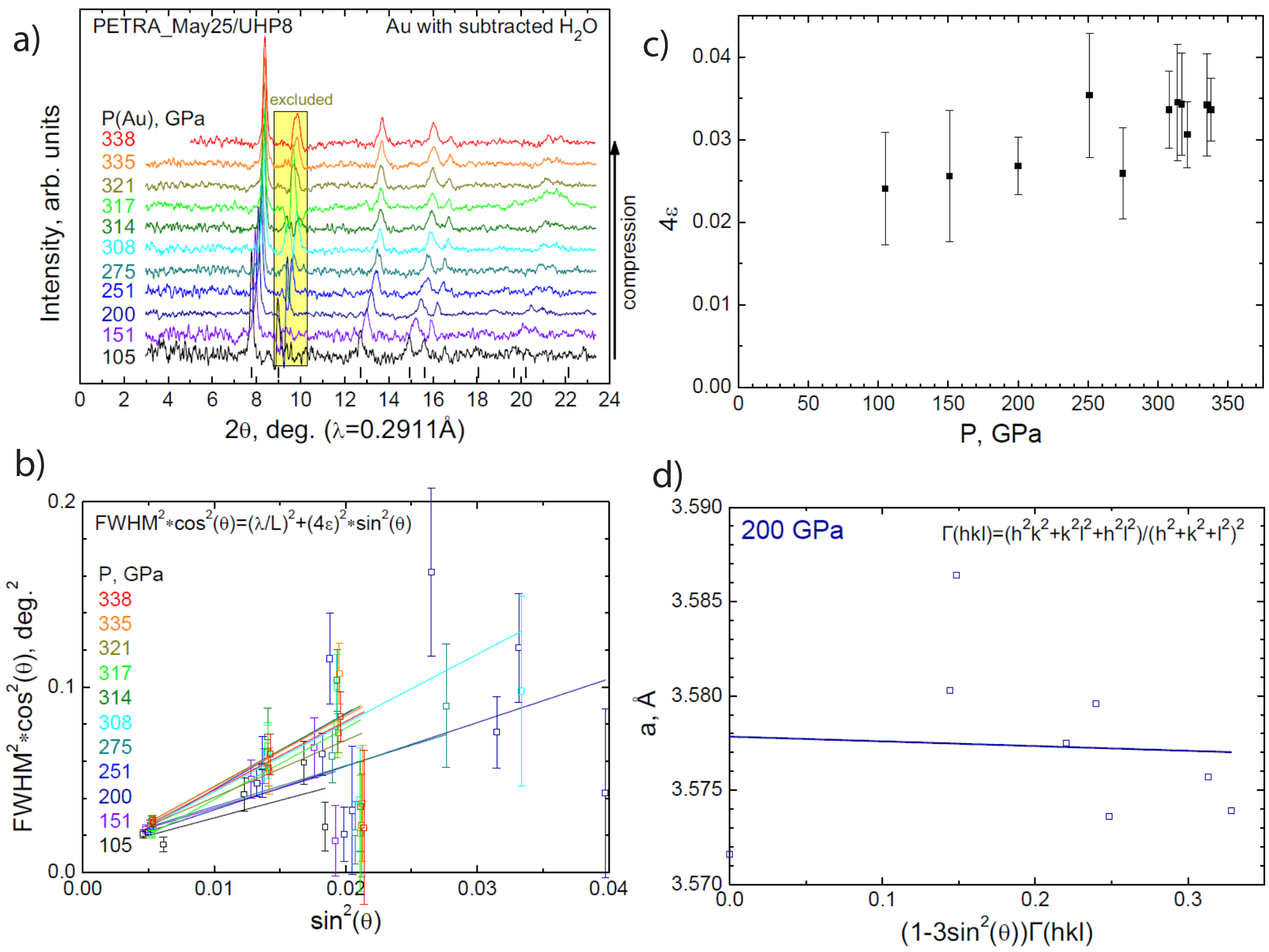

Supplementary Figure 8. a) Analysis of deviatoric stress in the UHP8 compression run based on the angular dependence of the FWHM of the x-ray diffraction peaks of the gold pressure marker. Representative x-ray powder diffraction patterns of gold upon compression from 105 to 338 GPa. Shaded yellow box indicates the (200) diffraction peak, which has been excluded from the analysis because of the overlap with the main peak group of ice. b) The full width half maximum (FWHM) of the observed diffraction peaks plotted as a function of $\sin^2\theta$ angle. The dependencies are fitted with a linear function and the resulting slopes gave us the strain 4ε. c) The strain 4ε, plotted as a function of pressure. d) Analysis of deviatoric stress in the UHP8 compression run based on the angular dependence of x-ray diffraction peak shift of the gold pressure marker at 200 GPa. The line shows the linear fit of the lattice parameters, calculated from the positions of individual peaks

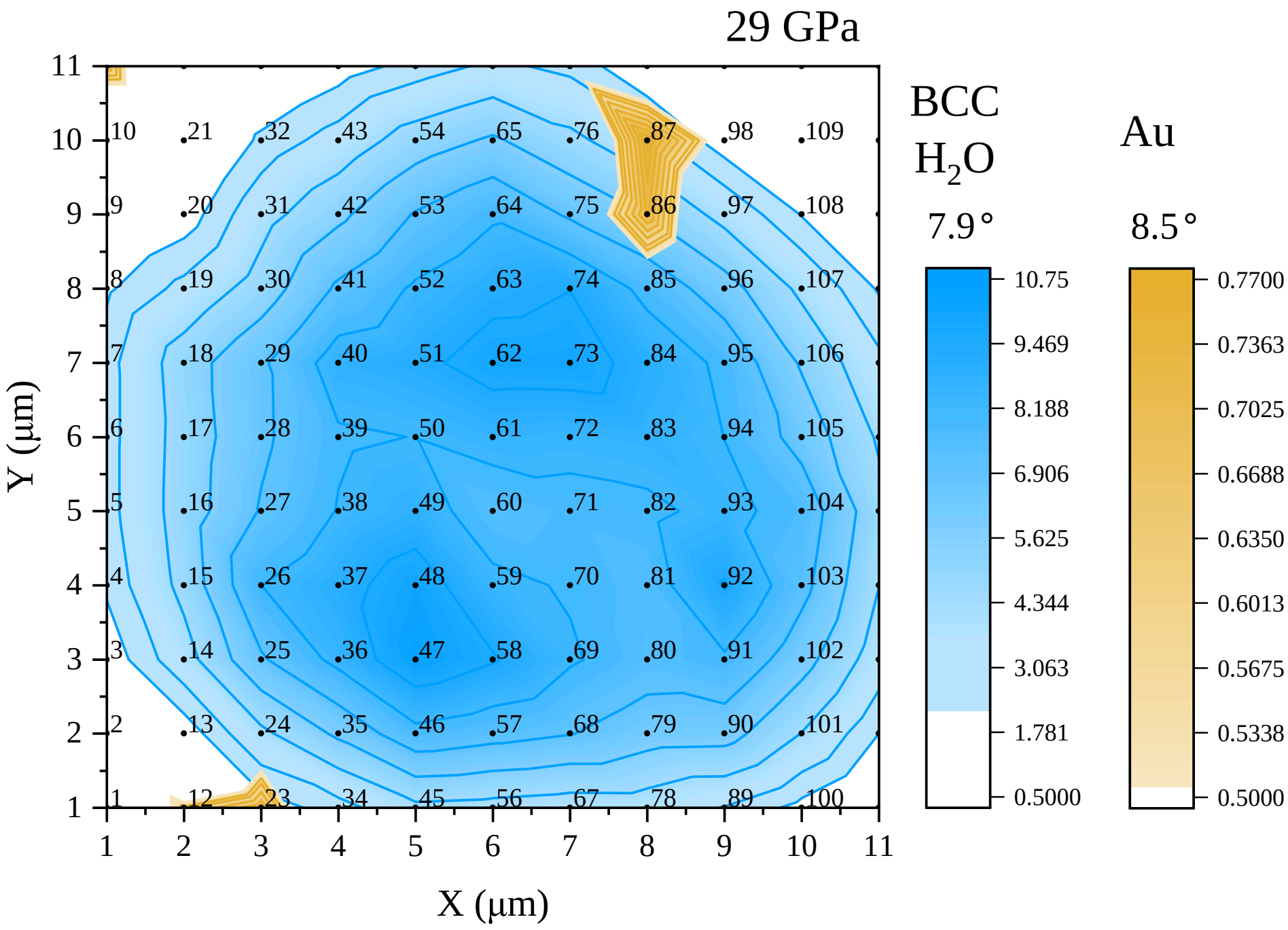

Supplementary Figure 9. Contour plot of the XRD mapping of the sample chamber, 2 μm step size, from experimental run UHP1, corresponding to the blue symbols in the top panel of Supplementary Figure 12 and red symbols in the bottom panel of Supplementary Figure 12. Blue regions indicate diffraction from ice where the (110) reflections of ice X are identified. Yellow regions correspond to diffraction peaks from Au, where the (111) reflections. Data were collected using a monochromatic X-ray beam with wavelength $\lambda = 0.29163$ Å.

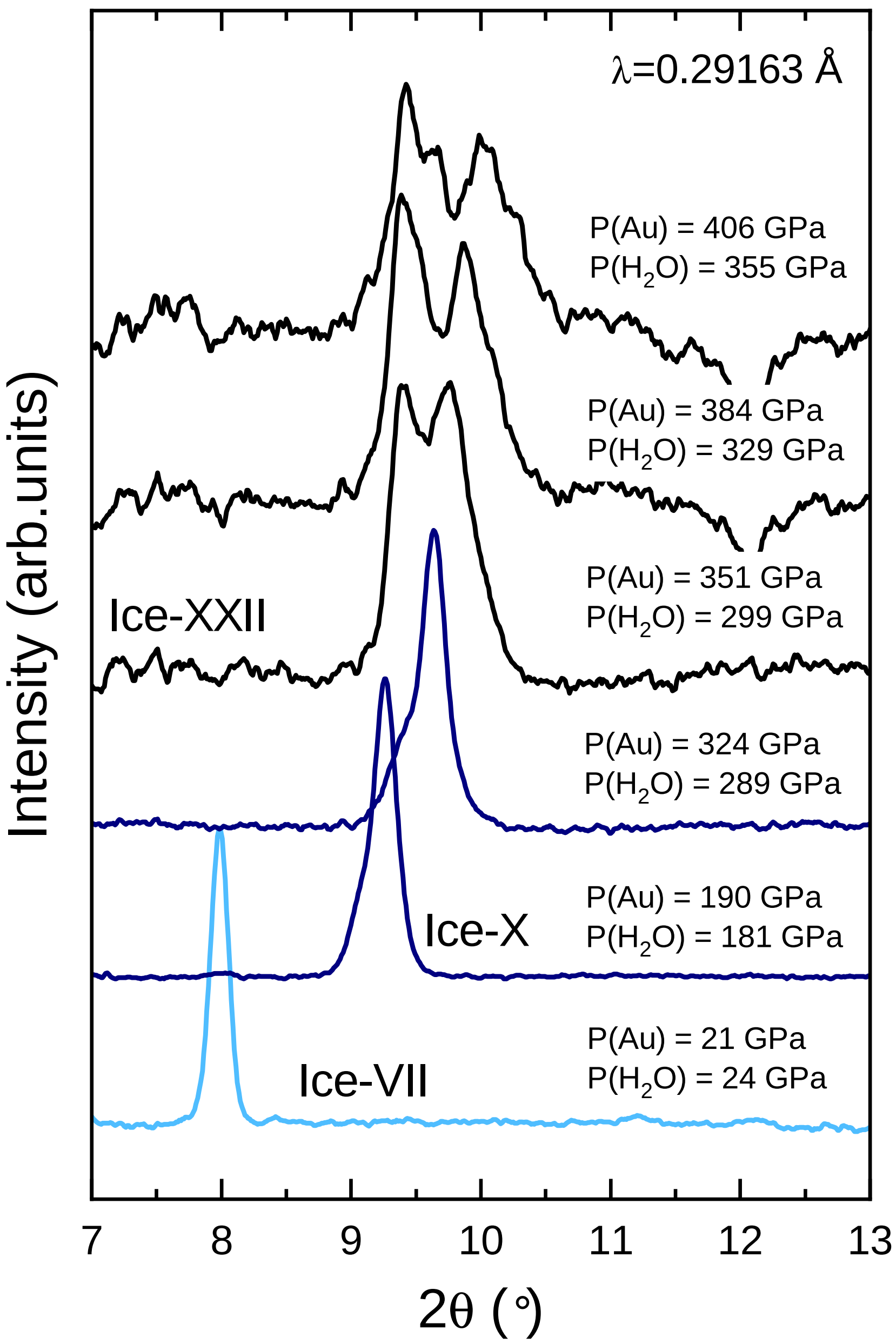

Supplementary Figure 10. Representative x-ray diffraction patterns upon compression from 21 GPa to 406 GPa, experimental run UHP1. Phase VII is represented by light blue, phase X by dark blue and phase XXII by black. Pressures are taken from Au[11] which was bridging between the gasket and the diamond anvil. As such the pressures are overestimated. The corrected pressures according to the ice equation of state are also given.

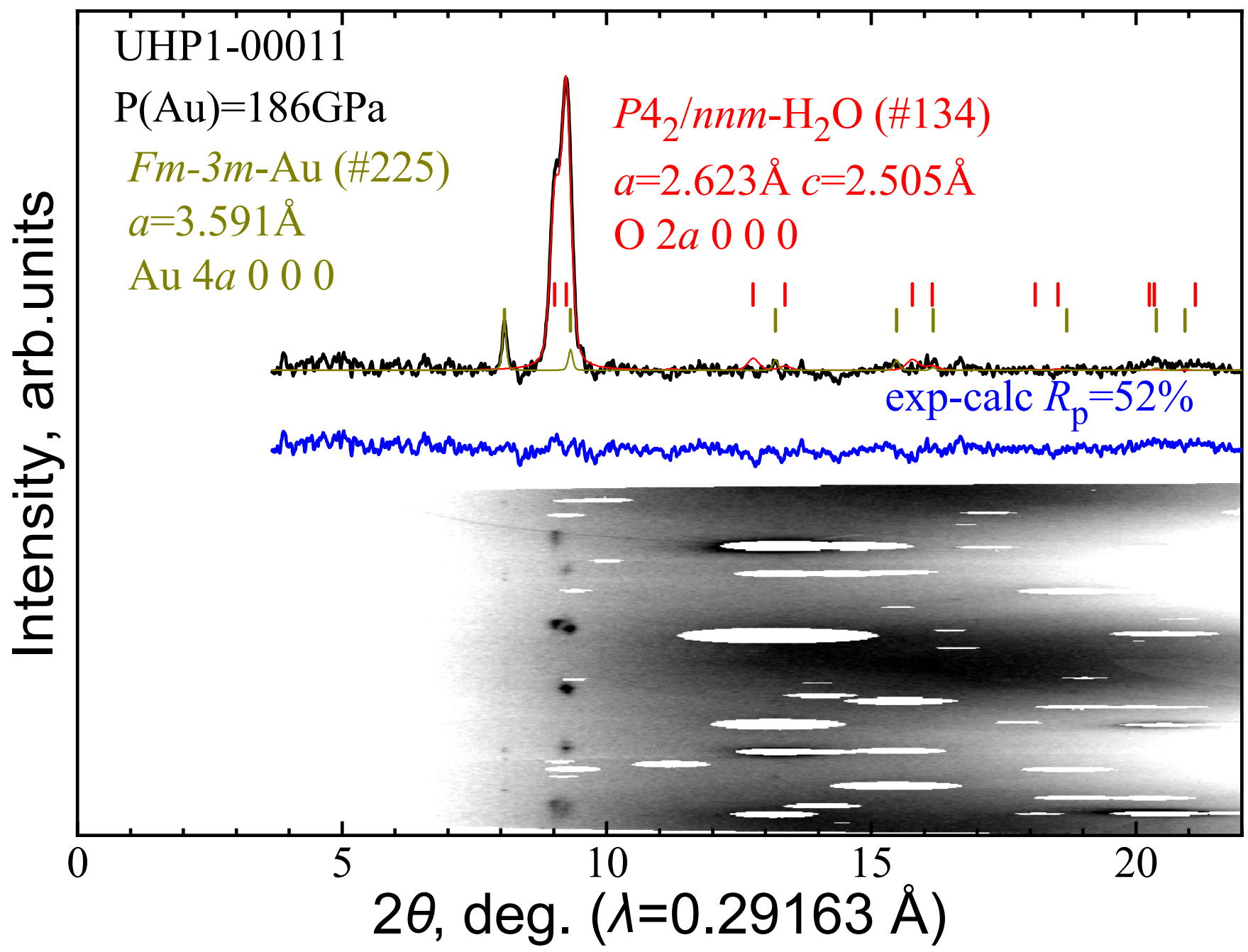

Supplementary Figure 11. Representative x-ray powder diffraction pattern of H_2O (black curve) at 186 GPa, together with the results of the Rietveld refinement of the structural model for $P4_2/nnm$ (red line). Experimental data are from the experimental run UHP1 and are shown in black. Gold is used as pressure marker and is as well refined (dark yellow). The refinement residuals are shown in blue. Inset below the residuals is the corresponding 2D diffraction cake image.

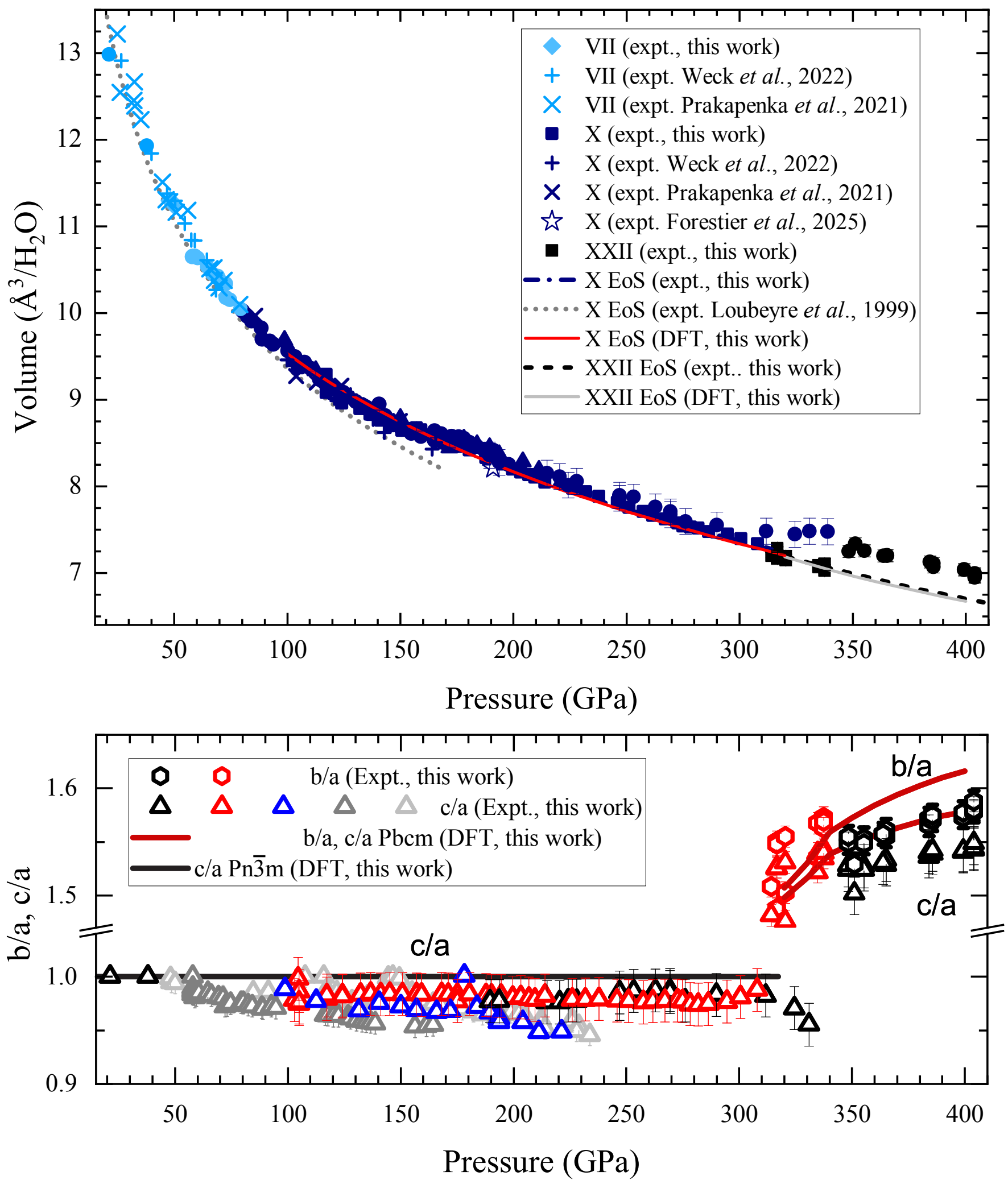

Supplementary Figure 12. Top:Volume as a function of pressure for the observed ice phases. Light blue symbols represent ice phase VII, dark blue symbols represent phase X and black symbols represent ice XXII. The experimental equation of states of ice X and ice XXII are represented as dotted-dash dark blue, and dashed black lines, respectively, see Table S2 for details. The solid lines represent the DFT P-V equation of states of ice X (red) and phase XXII (light gray). The grey dotted line shows the previously reported extrapolated equation of state of ice [6]. Previous experiments by Weck, et al.,[12], Prakapenka, et al.,[13] and Forestier, at al.,[14], are shown as '+', '×', and '⋆', respectively. The data scatter is due to the pressure gradients. Bottom: The b/a (hexagons symbols) and c/a ratios (triangle symbols) for phase X and phase XXII. Red symbols correspond to the experimental run UHP1, light grey to the run UHP3, dark gray to the run V4, black to the run UHP8, and blue to UHP5, respectively. The solid black line is the DFT calculated ratio for $Pn\bar{3}m$ ice X and red lines represent the DFT calculated b/a and c/a ratios for $Pbcm$ ice XXII.

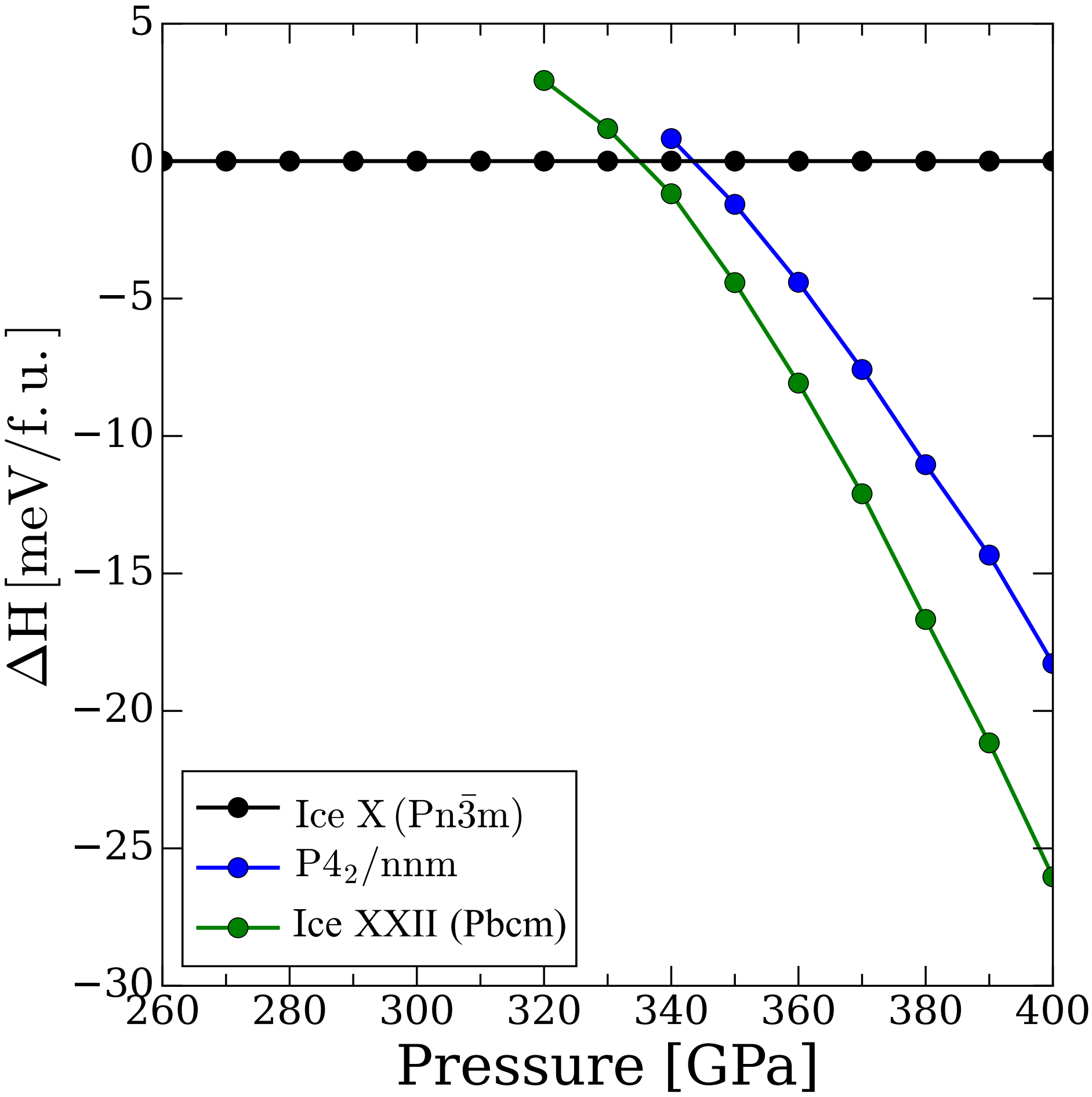

Supplementary Figure 13. Relative enthalpies of $Pn\bar{3}m$ ice X, $P4_2/nnm$, and $Pbcm$ calculated with the BLYP functional. The tetragonally elongated $P4_2/nnm$ structure as predicted by [15], is lower in energy than cubic ice X above the onset of stability for $Pbcm$ and does not become favoured energetically over $Pbcm$. At pressures below the ranges plotted for $P4_2/nnm$ and $Pbcm$ these structures transform spontaneously back to the cubic $Pn\bar{3}m$ structure in geometry optimization calculations.

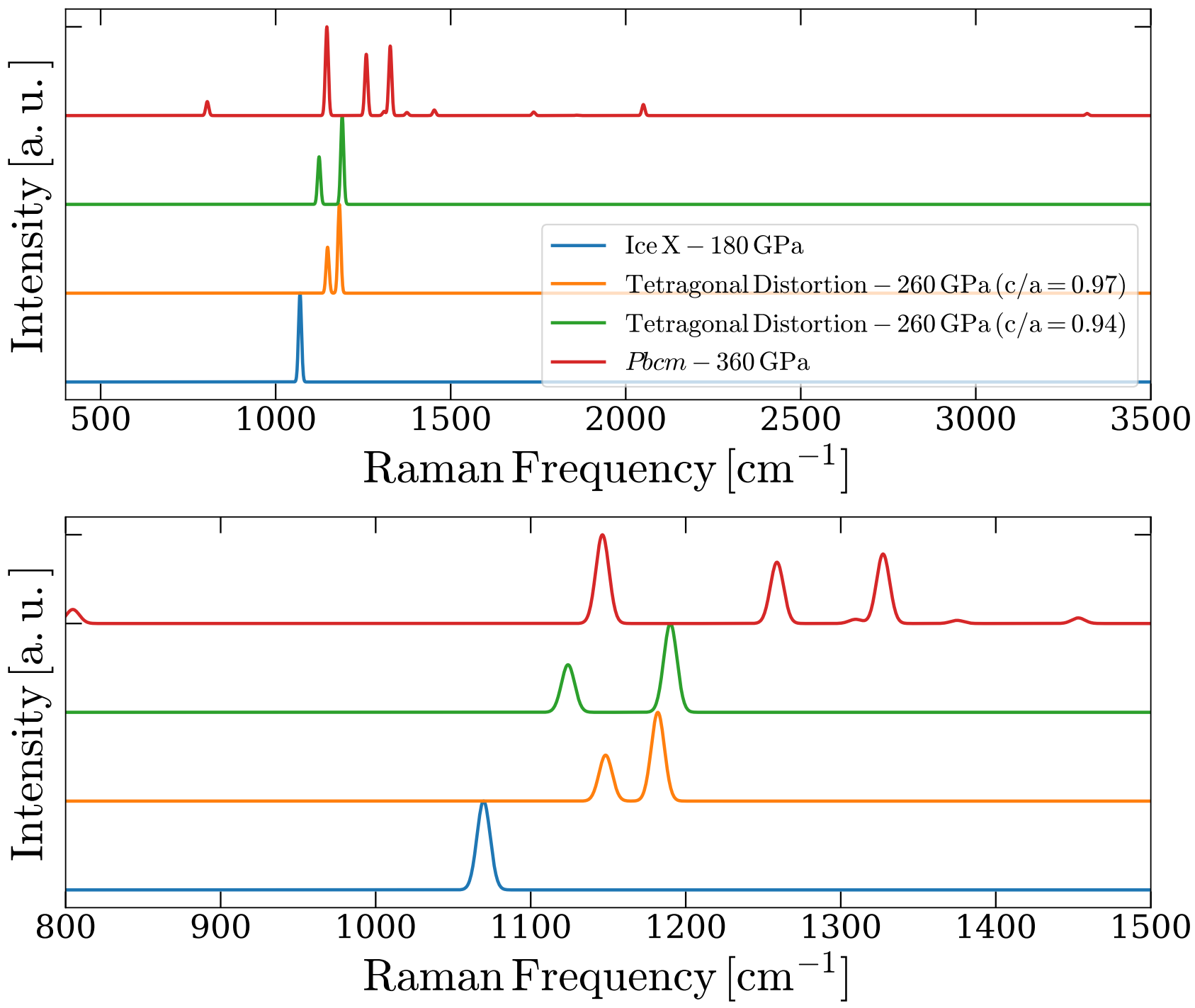

Supplementary Figure 14. Example Raman spectra from DFT at different pressures for $Pn\bar{3}m$ ice X, *Pbcm* ice XXII, and tetragonally distorted ice X. Top (bottom) panel show the entire (selected) frequency range. A Gaussian broadening of 10 cm^{-1} is applied to the DFT spectra for illustrative purposes. Volume-conserving tetragonal distortions of $Pn\bar{3}m$ ice X result in a peak splitting of its single Raman mode. Increased distortions increase the magnitude of the splitting approximately linearly for distortions up to 5% of the c-axis, corresponding to $c/a = 0.99 \ldots 0.93$. At 260 GPa the splitting for distorted cells with c/a =0.97 (0.94) is 33.7 (66.0) cm^{-1}.

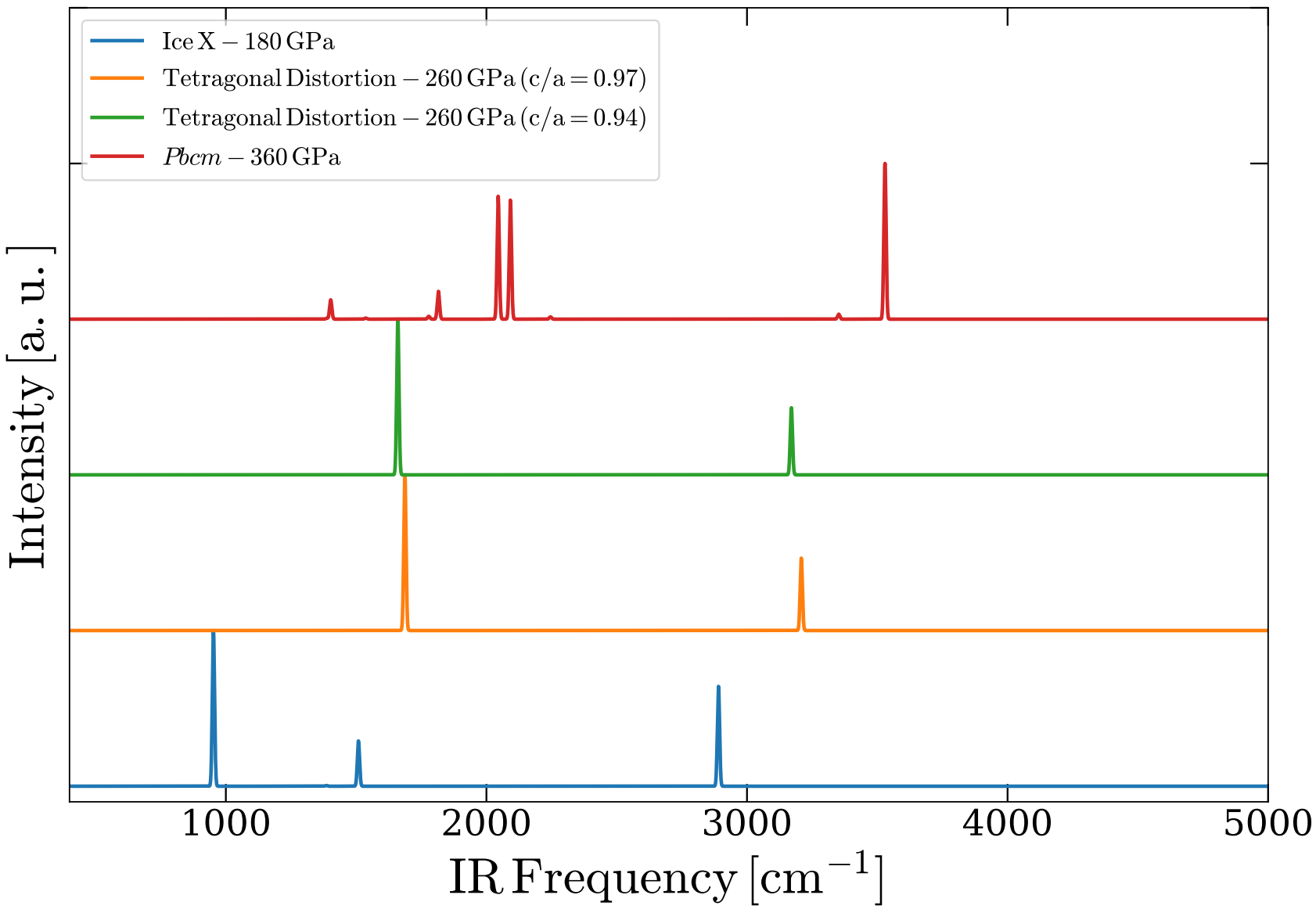

Supplementary Figure 15. Example IR spectra from DFT at different pressures for $Pn\bar{3}m$ ice X, $Pbcm$ ice XXII, and tetragonally distorted ice X. A Gaussian broadening of 10 cm^{-1} is applied to the DFT spectra for illustrative purposes. Volume-conserving tetragonal distortions of $Pn\bar{3}m$ ice X result in a redshift of the main IR modes in the 1500–2000 cm^{-1} and 3000–3500 cm^{-1} regions.

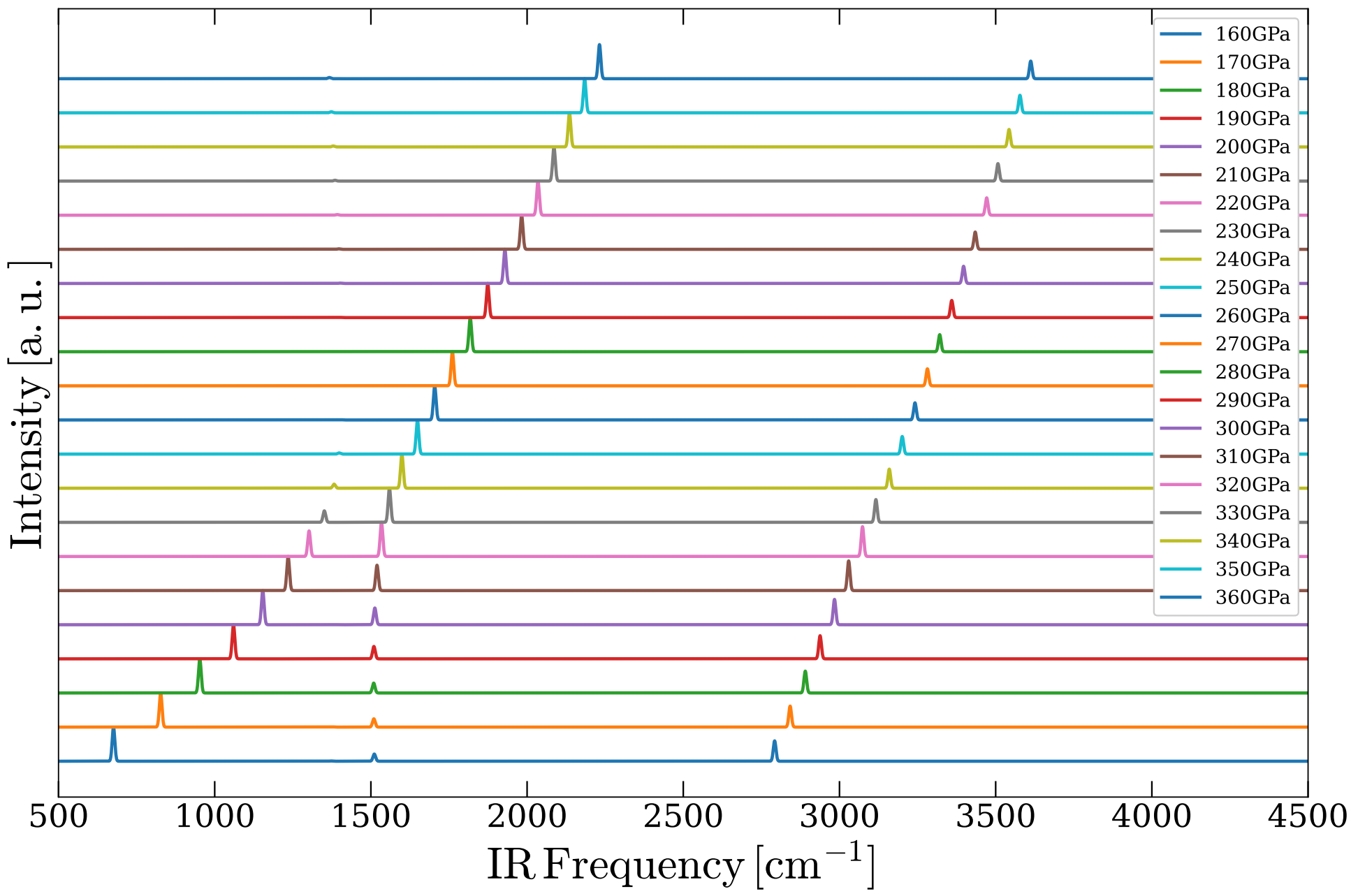

Supplementary Figure 16. IR spectra from DFT between 100 GPa and 360 GPa for $Pn\bar{3}m$ ice X. A Gaussian broadening of 10 cm^{-1} is applied to the DFT spectra for illustrative purposes. Increased pressure changes the predicted IR relative intensities. At 250 GPa the lowest frequency has dropped in intensity leaving only two main IR modes.

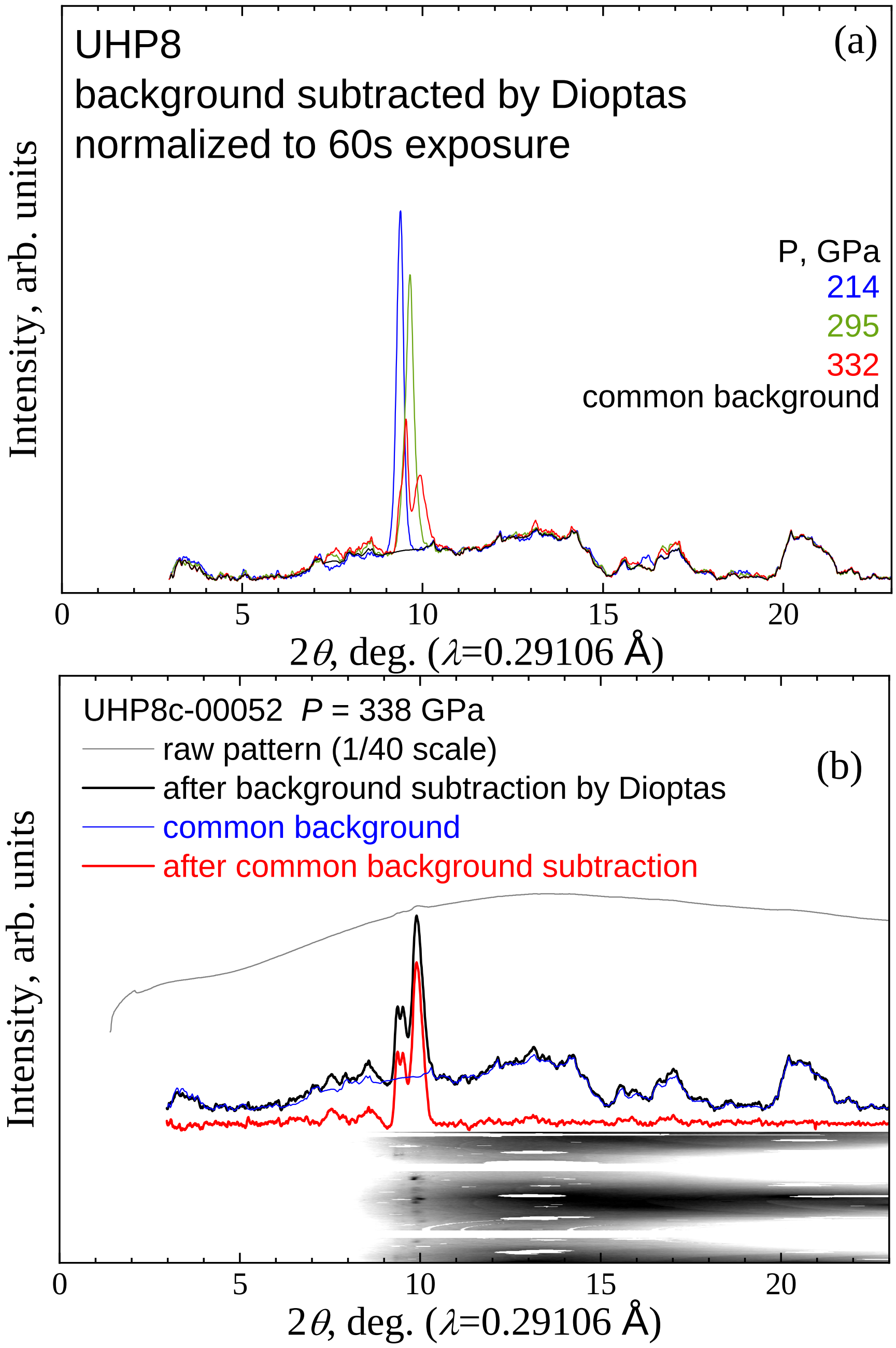

Supplementary Figure 17. Representative x-ray diffraction patterns from experiment UHP8. Top: diffraction patterns at 214 GPa (blue), 295 GPa (green), and 332 GPa (red). The common background to all three measurements is shown in black. Bottom: diffraction pattern at 338 GPa. The raw pattern (scaled by 1/40) is shown in gray, the applied common background in blue, and the resulting pattern after background subtraction in red. The grayscale insert shows the corresponding 2D diffraction-cake image.

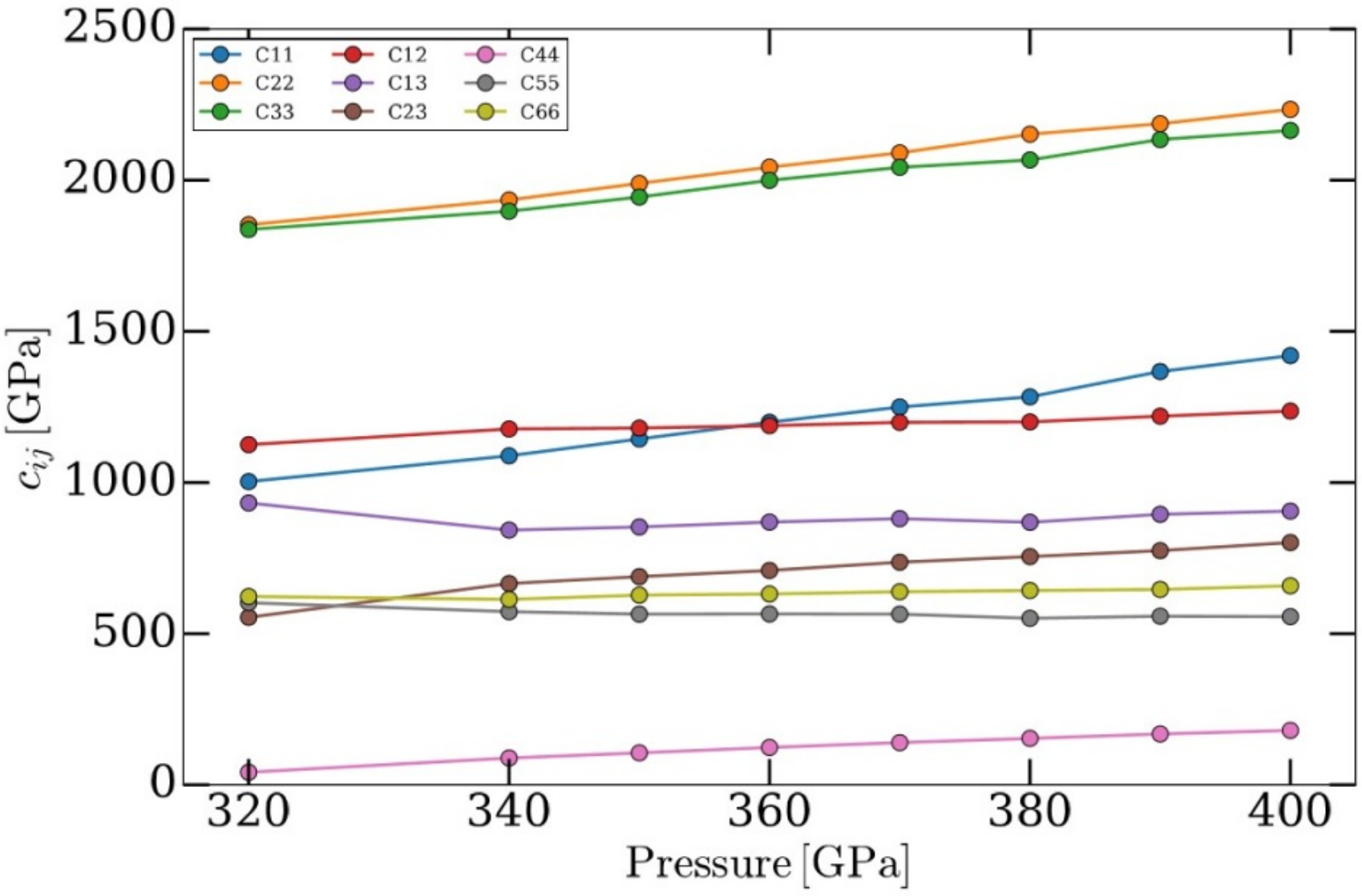

Supplementary Figure 18. The elastic constants as a function of pressure for *Pbcm* ice (XXII). All of the elastic constants are positive, and at all pressures fulfill the Born stability criteria. Hence, *Pbcm* ice is stable under non-hydrostatic pressure conditions. As c_{44} approaches zero at 300-320 GPa, *Pbcm* will revert back to ice X below those pressures.

Supplementary References

[1] Singh, A. K. & Kenichi, T. Measurement and analysis of nonhydrostatic lattice strain component in niobium to 145 gpa under various fluid pressure-transmitting media. *Journal of Applied Physics* **90**, 3269–3275 (2001). URL https://doi.org/10.1063/1.1397283.

[2] Wolanin, E. *et al.* Equation of state of ice vii up to 106 gpa. *Phys. Rev. B* **56**, 5781–5785 (1997). URL https://link.aps.org/doi/10.1103/PhysRevB.56.5781.

[3] Singh, A., Liermann, H. & Saxena, S. Strength of magnesium oxide under high pressure: evidence for the grain-size dependence. *Solid State Communications* **132**, 795–798 (2004). URL https://www.sciencedirect.com/science/article/pii/S0038109804008154.

[4] Hemley, R. J. *et al.* Static compression of H2O-ice to 128 GPa (1.28 Mbar). *Nature* **330**, 737–740 (1987). URL https://doi.org/10.1038/330737a0. ISSN 1476-4687.

[5] Sugimura, E. *et al.* Compression of h_2o ice to 126 gpa and implications for hydrogen-bond symmetrization: Synchrotron x-ray diffraction measurements and density-functional calculations. *Phys. Rev. B* **77**, 214103 (2008). URL https://link.aps.org/doi/10.1103/PhysRevB.77.214103.

[6] Loubeyre, P., LeToullec, R., Wolanin, E., Hanfland, M. & Hausermann, D. Modulated phases and proton centring in ice observed by x-ray diffraction up to 170 GPa. *Nature* **397**, 503–506 (1999). URL http://doi.org/10.1038/17300.

[7] Journaux, B. *et al.* Elasticity and dislocations in ice X under pressure. *Physics of the Earth and Planetary Interiors* **236**, 10–15 (2014). URL https://www.sciencedirect.com/science/article/pii/S0031920114001885.

[8] Tsuchiya, J. & Tsuchiya, T. First principles calculation of the elasticity of ice VIII and X. *The Journal of Chemical Physics* **146**, 014501 (2017). URL https://pubs.aip.org/jcp/article/146/1/014501/280485/First-principles-calculation-of-the-elasticity-of.

[9] Gambin, D., Dudek, K. K., Dudek, M. R., Grima, J. N. & Gatt, R. The mechanical properties of ice X with particular emphasis on its auxetic potential. *Journal of Physics and Chemistry of Solids* **150**, 109717 (2021). URL https://www.sciencedirect.com/science/article/pii/S0022369720312749.

[10] Goncharov, A. F., Struzhkin, V. V., Mao, H.-k. & Hemley, R. J. Raman spectroscopy of dense H_2O and the transition to symmetric hydrogen bonds. *Phys. Rev. Lett.* **83**, 1998–2001 (1999). URL https://link.aps.org/doi/10.1103/PhysRevLett.83.1998.

[11] Dorfman, S. M., Prakapenka, V. B., Meng, Y. & Duffy, T. S. Intercomparison of pressure standards (Au, Pt, Mo, MgO, NaCl and Ne) to 2.5 Mbar. *Journal of Geophysical Research: Solid Earth* **117**, B08210 (2012).

[12] Weck, G. *et al.* Evidence and stability field of fcc superionic water ice using static compression. *Phys. Rev. Lett.* **128**, 165701 (2022). URL https://link.aps.org/doi/10.1103/PhysRevLett.128.165701.

[13] Prakapenka, V. B., Holtgrewe, N., Lobanov, S. S. & Goncharov, A. F. Structure and properties of two superionic ice phases. *Nature Physics* **17**, 1233–1238 (2021). URL https://doi.org/10.1038/s41567-021-01351-8.

[14] Forestier, A. *et al.* X-ray signature of the superionic transition in warm dense fcc water ice. *Phys. Rev. Lett.* **134**, 076102 (2025). URL https://link.aps.org/doi/10.1103/PhysRevLett.134.076102.

[15] Reinhardt, A. *et al.* Thermodynamics of high-pressure ice phases explored with atomistic simulations. *Nature Communications* **13**, 4707 (2022). URL https://doi.org/10.1038/s41467-022-32374-1.
